# Effect of L- to D-Amino Acid Substitution on Stability and Activity of Antitumor Peptide RDP215 against Human Melanoma and Glioblastoma

**DOI:** 10.3390/ijms22168469

**Published:** 2021-08-06

**Authors:** Theresa Maxian, Lisa Gerlitz, Sabrina Riedl, Beate Rinner, Dagmar Zweytick

**Affiliations:** 1 Institute of Molecular Biosciences, University of Graz, Humboldtstraße 50/III, A-8010 Graz, Austria; theresa.maxian@meduniwien.ac.at (T.M.); lisa.gerlitz@cbmed.at (L.G.); 2 Department of General Surgery, Medical University of Vienna, Währinger Gürtel 18-20, A-1090 Wien, Austria; 3 CBmed Biomarker Research, Stiftingtalstraße 5, A-8010 Graz, Austria; 4 BioHealth, A-8010 Graz, Austria; 5 BioTechMed-Graz, A-8010 Graz, Austria; beate.rinner@meduni-graz.at; 6 Center for Medical Research, Medical University of Graz, Stiftingtalstraße 24, A-8010 Graz, Austria

**Keywords:** antitumor peptides, D-amino acids, serum stability, melanoma, glioblastoma

## Abstract

The study investigates the antitumor effect of two cationic peptides, R-DIM-P-LF11-215 (RDP215) and the D-amino acid variant 9D-R-DIM-P-LF11-215 (9D-RDP215), targeting the negatively charged lipid phosphatidylserine (PS) exposed by cancer cells, such as of melanoma and glioblastoma. Model studies mimicking cancer and non-cancer membranes revealed the specificity for the cancer-mimic PS by both peptides with a slightly stronger impact by the D-peptide. Accordingly, membrane effects studied by DSC, leakage and quenching experiments were solely induced by the peptides when the cancer mimic PS was present. Circular dichroism revealed a sole increase in β-sheet conformation in the presence of the cancer mimic for both peptides; only 9D-RDP215 showed increased structure already in the buffer. Ex vitro stability studies by SDS-PAGE as well as in vitro with melanoma A375 revealed a stabilizing effect of D-amino acids in the presence of serum, which was also confirmed in 2D and 3D in vitro experiments on glioblastoma LN-229. 9D-RDP215 was additionally able to pass a BBB model, whereupon it induced significant levels of cell death in LN-229 spheroids. Summarized, the study encourages the introduction of D-amino acids in the design of antitumor peptides for the improvement of their stable antitumor activity.

## 1. Introduction

At present, cancer is a leading cause of death worldwide (https://gco.iarc.fr/today/home, 1 November 2020). The Global Cancer statistics 2020 estimates that 19.3 million patients have been newly diagnosed with cancer and that the disease is responsible for about 10.0 million deaths in 2020 [1]. Since the development of the first treatment possibilities as surgery and radiation, enormous advances have been made through chemotherapy, targeted therapy and combinations thereof [2]. Nevertheless, cancer therapy is not always effective due to the resistance or existence of non-reachable metastases. It can further lead to severe side effects owing to a lack of specificity against tumor cells. Thus, some cancer types still show poor treatability and prognosis, e.g., malignant melanoma and glioblastoma, cancers to the skin and brain [3,4].

The cutaneous form of melanoma is common in the Western world and causes 75% of deaths related to skin cancer [5]. Early detection of primary melanoma is crucial for preventing a metastatic spread and subsequently lowering the disease’s mortality [6]. Additionally, chemoresistance of malignant melanoma only manifests a median overall survival of 6–10 months [7]. Until the last decade, the common therapy included surgical resection, chemotherapy with the alkylating agent dacarbazine (DITC) and interleukin (IL-2) in high doses [8]. The combination of these therapies only resulted in moderate responses in patients with a metastatic disease. Though, a better understanding of both mutations driving tumorigenesis and immune escape mechanisms contributed to the development of new drugs in the form of targeted therapies [7,8]. Two recent available therapeutic strategies are able to enhance the prognosis for patients with melanoma [5], which are the introduction of immune-checkpoint inhibitors in cytotoxic T lymphocyte-associated antigen 4 (CTLA-4) and programmed death 1 (PD-1) enabling a reactivation of the immune responses against cancer [5,7]. The second strategy uses BRAF (V-Raf murine sarcoma viral oncogene homolog B1) and MEK (Mitogen-activated protein kinase kinase) inhibitors to block the mitogen-associated protein kinase (MAPK) pathway that is constitutively activated by BRAF V600 mutations in 45% of melanoma patients [5,7]. However, despite this enormous progress in the treatment of melanoma, most patients still develop primary or secondary resistance [7]. Furthermore, there is no treatment of the most aggressive forms of melanoma, including brain metastases and metastases from ocular melanoma [7].

Another cancer with poor treatability and prognosis, glioblastoma (GBM), is one of the most aggressive types of gliomas with grade number IV. GBM can occur de novo, resulting in primary GBMs or can derive from a lower-grade tumor being classified as secondary GBMs [9,10]. Malignant gliomas infiltrate into normal brain parenchyma, leaving restricted therapeutic strategies by causing nonspecific damage to the surrounding of normal brain tissue [11]. Despite the standard treatment of surgical removal followed by radiation and chemotherapy with the alkylating agent temozolomide (TMZ), GBM remains incurable with a median overall survival of 12–15 months after diagnosis [9]. Further, in the case of patients with increased DNA repair, TMZ is less effective, also being predicted with poor prognosis [12]. The monoclonal antibody bevacizumab that inhibits the growth of tumor blood vessels by binding to VEGF (vascular endothelial growth factor) prolongs overall survival by 4 months but only in recurrent, not in primary glioblastoma [13]. The highly invasive tumor cells are responsible for tumor recurrence due to their ability to bypass surgical resection and resistance to conventional treatment [12]. Thus, even upon application of all possible therapeutic options, the tumor still tends to reoccur in 70% of GBM patients within one year of diagnosis [12], and less than 5% survive more than five years upon diagnosis [14,15]. Although global incidence rates are less than 10 per 100,000, the low survival rates and the fact of being the third most cause of cancer deaths between the age of 15 and 34 years makes this cancer type a crucial health issue [16,17]. 

Therefore, new and optimized therapies are needed, specifically targeting glioma and melanoma cells while leaving the neighboring non-malignant tissue unharmed and possessing the ability to pass through the blood-brain barrier (BBB), which a majority of drugs fail to, so far [18]. Several new key approaches for brain targeting include physiological transport mechanisms such as adsorptive-mediated transcytosis, inhibition of active efflux pumps, receptor-mediated transport, cell-mediated endocytosis or the use of peptide vectors [19]. Indeed, newer approaches have shown that peptides as RVG29 [20] or apamin [21] can cross the barrier and can therefore be used as so-called Trojan horses to shuttle other (larger) compounds to the brain. The latter is derived from bee venom and has been modified to be applied without toxic side effects [21]. 

Cationic antitumor peptides derived from the human host defense peptide Lactoferricin [22] studied in our lab (PCT/EP2014/050330; US 14/760,445; EP 14700349.5) have been shown to selectively kill cancer cells of different types, including malignant melanoma and their metastases [23,24,25,26] and glioblastoma [24] in 2D and 3D [26] in vitro and in vivo [25] by targeting the negatively charged lipid phosphatidylserine (PS), specifically exposed by cell membranes of cancer cells [27,28]. Cell death appeared by peptide-induced apoptosis [24], a process normally blocked, e.g., in melanoma by inhibition of Apaf-1 [29]. Several highly active and specific peptide derivatives with about 5- to 25-fold specificity for melanoma over normal dermal fibroblasts or melanocytes were designed with a similar pattern of a secondary structure comprising a mostly proline induced loop in the middle of the peptide flanked by two helices or two β-strands and a net charge of +9 and above [24,26]. 

One of these peptides, R-DIM-P-LF11-322, exhibits selective activity against the cancer cell marker PS on model systems and cytotoxicity against melanoma, glioblastoma and rhabdomyosarcoma without significant effect on non-neoplastic cells as melanocytes or fibroblasts at antitumor-active concentrations [23,24,26]. Through the deletion of two Phe residues, hydrophobicity and slight cytotoxicity were decreased, resulting in a still nicely active peptide R-DIM-P-LF11-334 [25]. It was demonstrated that R-DIM-P-LF11-334, in addition to its specific activity on PS in model systems and cancer-specific toxicity on malignant melanoma in vitro, also induces tumor regression of human malignant melanoma in mouse xenografts in vivo [25]. Though, in the study, a serum-induced destabilizing effect on the peptide was observed [25]. Therefore, further optimization regarding proteolytic stability was performed. In a recent study, Grissenberger et al. reported about (retro) di-peptide R-DIM-P-LF11-215 (RDP215) with a net charge of +9 and moderate hydrophobicity that exhibited the highest specific antitumor activity of the peptide derivatives in 2D and 3D in vitro studies so far [26], which might potentially be due to an increase in stability. 

Indeed, the most challenging aspects in the development of successful therapeutic peptides is metabolic instability, short half-life and, consequently, limited residence time and concentration in tumor tissue [30]. Proteases present in cells, tissues and body fluids, normally essential for the digestion of food (extracellular), post-translational processing and subcellular localization, are often degrading the potential peptide therapeutic [31]. The modification of the peptide termini, such as N-acetylation and C-amidation, is therefore helpful to prevent peptide degradation [30]. The latter is already applied for the peptides used in our lab. Another approach to enhance the stability of peptides appears through structural modifications and removal of potential cleavage sites of proteases as natural L-amino acids at the termini or as, e.g., Arg by substitution with their non-natural D-amino acid counterpart [30,31,32,33]. D-amino acids share identical chemical and physical characteristics with L-amino acids, except for the ability to rotate plane-polarized light in the opposite direction [34]. Further, unlike L-amino acids, they rarely act as a substrate of endogenous proteases since peptide bonds neighboring D-amino acids are not recognized by them [34]. Consequently, the improved stability of peptides containing D-amino acids results in longer in vivo circulation half-time [34]. 

The replacement of L-amino acids to their D-enantiomer has not only been shown to improve the serum stability but also the selective toxicity [32,34]. In 2004, Papo and Shai demonstrated that an amphipathic D-peptide, in contrast to its parental L-form, was not only able to reduce the tumor growth of various human prostate carcinoma xenografts in vivo when administered intratumorally but also showed increased cancer specificity in vitro and therefore potentially decreased side effects [35]. 

Therefore, this study aims to investigate the potential influence of D-amino acids on the proteolytic stability and selective antitumor activity of the Lactoferricin-derived peptide, R-DIM-P-LF11-215 (RDP215), and its D-amino acid variant, 9D-R-DIM-P-LF11-215 (9D-RDP215). A combination of liposomal model studies mimicking cancer and non-cancer membranes, ex vitro studies in the presence of different sera (fetal bovine serum and human serum), 2D in vitro studies with cell lines of malignant melanoma (A375, SBcl-2), 2D and 3D in vitro studies with glioblastoma (LN-229, U-87 mg) and a BBB model is applied to further optimize antitumor peptides as new, specific and potent cancer therapeutics.

## 2. Results

Within the study, the two LF11-derived cationic antitumor peptides, R-DIM-P-LF11-215 (RDP215) and D-amino acid variant 9D-R-DIM-P-LF11-215 (9D-RDP215), are characterized to determine the efficiency of D-amino acids in increasing proteolytic stability and selective toxicity for negatively charged PS exposed to melanoma and glioblastoma, two cancers with poor treatability and prognosis. Both peptides comprise the same sequence of amino acids (H-FWRIRIRR P RRIRIRWF-NH_2_); however, in the D-peptide, the L-amino acids Phe (F) and Arg (R) are substituted by their D-enantiomers (see Table 1).

### 2.1. In Silico Structure Prediction of RDP215 and 9D-RDP215

Avrahami et al. showed that amphipathic helical peptides, such as pardaxin, melittin or magainin, maintained their ability to interact with negatively charged phospholipid membranes after the replacement of L-amino acids with their D-enantiomers, further preserving their functional structure [36]. However, the incorporation of D-amino acids may influence the secondary structure of the peptide and consequently its interaction with membranes. Thus, in silico structure predictions using the online programs PEP-fold [37,38,39] and PEPstrMOD [40,41] and circular dichroism spectroscopy studies (Section 2.2.1) in the presence of model membranes were performed. 

**Figure 1:**
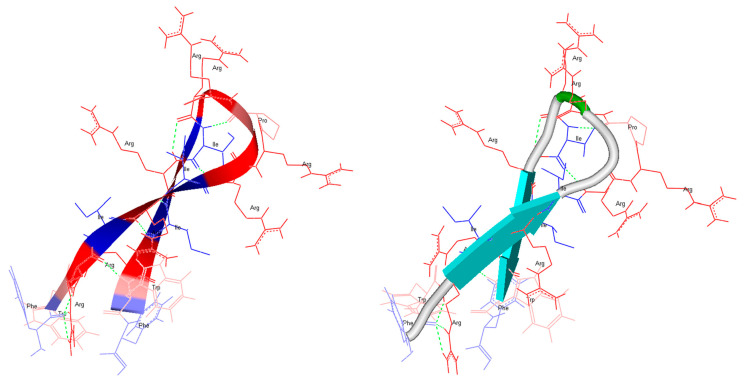
In silico structure prediction of RDP215 with PEP-fold. The amino acids are illustrated in black in the backbone. Left: Color code of positions of amino acids in backbone: Arg (red), Ile, Trp (blue) and Pro (pink). Right: The structure prediction for RDP215 suggests the conformation of a β-strand structure consisting of two β-strands (turquoise) divided by a loop region (green).

**Figure 2:**
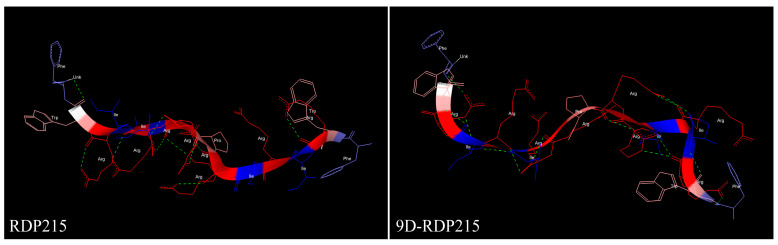
In silico structure prediction of RDP215 **(left**) and 9D-RDP215 (**right**) with PEPstrMOD. The PEPstrMOD structure prediction revealed a rather extended structure with similar distribution of amino acid residues for the peptides RDP215 and 9D-RDP215.

The potential impact of D-amino acids on the peptide structure was examined by in silico structure predictions using the online programs PEP-fold (Figure 1) and PEPstrMOD (Figure 2). The online program PEP-fold [37,38,39] predicted a structure for RDP215 with the conformation of two β-strands divided by a loop region (Figure 1). An amphipathic distribution of charged and hydrophobic amino acids with an agglomeration of exposed positively charged amino acid residues in the loop and hydrophobic amino acid residues at the termini sites can be observed.

Due to PEP-fold’s inability to evaluate D-enantiomers, the in silico structure of 9D-RDP215 could only be predicted using the program PEPstrMOD [40,41]. It shows a mostly extended structure for both RDP215 and 9D-RDP215 (Figure 2). The distribution of positively and hydrophobic amino acids is comparable. Though, it appears that the D-peptide comprises a higher number of hydrogen bridges (green dotted lines), which, in addition to the reduced accessibility for proteases, might cause an increase in stability and consequently in activity.

### 2.2. Effect of RDP215 and 9D-RDP215 on Liposomal Model Systems Mimicking Cancer and Non-Cancer Membranes

The cell surface of non-cancer cells exposes neutrally charged components, such as the zwitterionic phosphatidylcholine (PC) [42], whereas cancer plasma membranes, due to a loss of asymmetry, expose the negatively charged lipid phosphatidylserine (PS) to the outer, normally completely retained in the inner leaflet [27,43]. The negatively charged cancer membrane of, e.g., melanoma and glioblastoma, thus offers a specific target for cationic amphipathic peptides [27,28,44]. For analysis of a potential effect of D- amino acids on the interaction of peptides with cancer cells, liposomes mimicking cancer (PS) and non-cancer (PC) membranes were evaluated in the presence of RDP215 and 9D-RDP215.

#### 2.2.1. Membrane Induced Structure of RDP215 and 9D-RDP215

**Figure 3:**
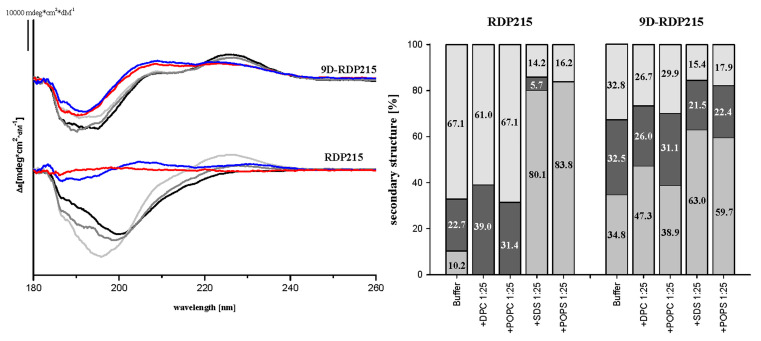
Circular Dichroism (CD) spectroscopy of RDP215 and 9D-RDP215. Investigation of peptide structures and their behavior in the presence of cancer mimics POPS/SDS and non-cancer mimics POPC/DPC. Left: CD spectra of RDP215 and 9D-RDP215 in sodium phosphate buffer (black lines) or the presence of DPC (light gray lines), POPC (dark gray lines), SDS (blue lines) and POPS (red lines) at peptide to surfactant ratios of 1:25. Right: Secondary structures calculated from CD spectra using Spectra secondary structure estimation [45]. The β-turns are shown in middle gray; turns are shown in dark gray; random coil structures are shown in light gray at the top. RDP215 and 9D-RDP215 are specific peptides and change their secondary structure only in the presence of the cancer mimics POPS and SDS. The substitution of D-amino acids does not alter the general structural conformation behavior of the peptides, though the structured part in solution is increased. Data represent the outcome of three independent experiments.

Selectivity and structural studies with RDP215 and 9D-RDP215 were performed using circular dichroism-spectroscopy (CD). POPS and POPC were used as cancer and non-cancer mimics, respectively. Since CD-experiments in a lipidic environment, such as POPS or POPC, often deliver rather noisy data, SDS was also taken as micellar negatively charged cancer and DPC as the neutral non-cancer mimic. In all cases, a peptide-to-lipid ratio of 1:25 was used. CD-spectroscopy data (Figure 3) revealed that RDP215 is mainly unstructured in the solution (about 70%), exhibiting low ratios of β-sheet conformation (10%) and turns to a similar extent. In the presence of the non-cancer mimics DPC and POPC, RDP215 remains overall unstructured (60%–70%) comprising turns at the extent of about one-third. In contrast, 9D-RDP215 appears already majorly structured in the solution, exhibiting 35% ß-sheet conformation. In the presence of the non-cancer mimics DPC and POPC, the D-peptide 9D-RDP215 and the L-peptide RDP215 show a similar structural behavior as in the solution. However, in the presence of the cancer mimics SDS and POPS, both peptides, RDP215 and 9D-RDP215, reveal a significant increase in the ratio in the ß-sheet structure, which is 80% more pronounced within the L-peptide. The results indicate that the peptides specifically change their structure in the presence of the negatively charged cancer cell mimics, SDS and POPS, and presumably in the presence of the cancer cell membrane itself. This change of structure might cause a specific interaction with the cancer membrane. Overall, both peptides show similar structural behavior, although, the increased structure of the D-peptide already in the solution might influence the kinetics and efficiency of the interaction of 9D-RDP215 with its potential target PS on cancer cells.

#### 2.2.2. Effect of D-Amino Acids on Membrane Permeabilization

**Figure 4:**
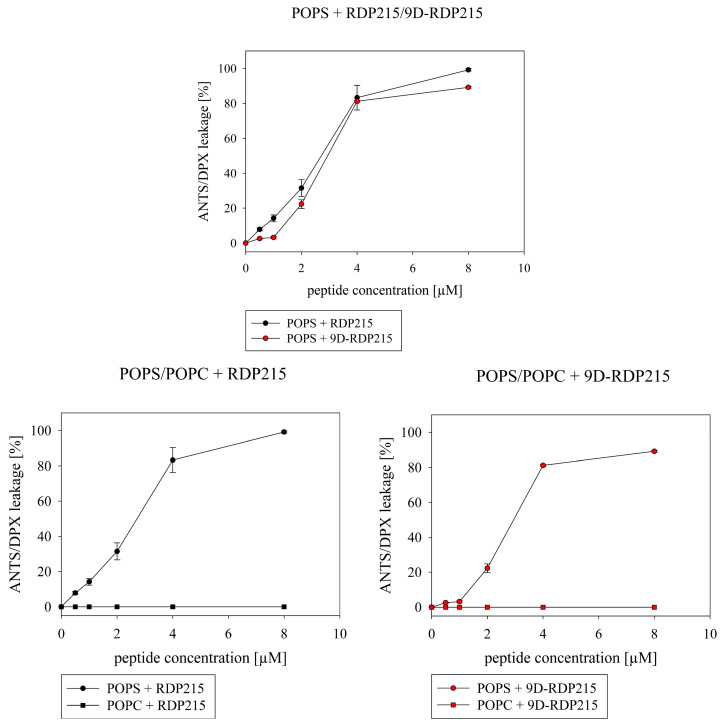
Membrane permeabilization of RDP215 and 9D-RDP215 on liposomes composed of cancer mimic POPS and non-cancer mimic POPC in ANTS/DPX leakage studies. (top). The leakage of POPS liposomes in the presence of RDP215 and 9D-RDP215. Bottom; left: The leakage of POPS and POPC liposomes in the presence of RDP215. Bottom; right: The leakage of POPS and POPC liposomes in the presence of 9D-RDP215. Mean values are given with standard deviation from at least 3 independent experiments. Both peptides show strong interaction only with the cancer mimic PS. At 8 µM peptide concentration, 99.2 ± 0.7% (RDP215, black circles) and 89.2 ± 0.6% (9D-RDP215, red circles) of ANTS/DPX is released from the POPS liposomes compared to no leakage induced on POPC liposomes (RDP215, black squares/9D-RDP215, red squares). Data represent medium values with standard deviations given of at least three independent experiments.

Leakage studies were performed to assess the potentially different effects of RDP215 and 9D-RDP215 on membrane permeabilization of liposomal cancer and non-cancer mimics. As depicted in Figure 4, the addition of increasing amounts (0.5–8 µM) of the two peptides to POPS liposomes mimicking cancer cells resulted in high and comparable membrane permeabilization shown by increased release of ANTS/DPX (Figure 4 top). Already at 4 µM peptide concentration, both peptides induce approximately 80% of the ANTS/DPX release of POPS liposomes (Table 2). With 90%, the D-peptide induces slightly decreased and slower membrane permeabilization by about 10% less than RDP215 at 8 µM. Figure 4 (bottom) shows that, in contrast to the strong membrane permeabilization of the cancer mimic POPS (Table 2), no permeabilization of the “healthy” mimic POPC is observed at any concentration studied (0.5–8 µM) (Figure 4 bottom; left and right). This indicates a strong and specific permeabilizing effect of both peptides for cancer membranes without effects on healthy cells.

#### 2.2.3. Effect of D-Amino Acids on Membrane Perturbation and Destabilization

**Figure 5:**
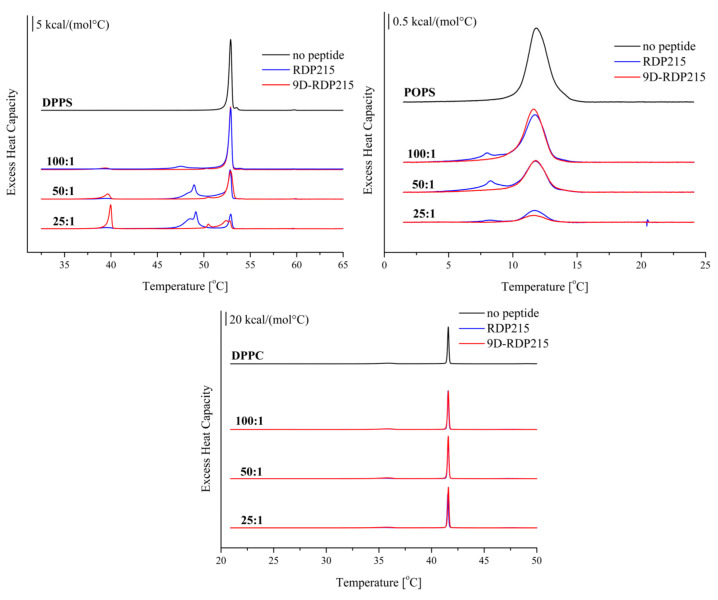
Thermotropic phase behavior of liposomes composed of DPPS, POPS and DPPC in the absence and presence of RDP215 and 9D-RDP215. Liposomes of DPPS (top; left) and POPS (top; right) were used to determine the effect of the two peptides on the cancer marker PS (lipid to peptide molar ratios 100:1, 50:1 and 25:1). The effect on liposomes of the non-cancer mimic DPPC is shown at the bottom. The thermograms in black represent the thermotropic phase behavior of pure lipid in the absence of peptides, blue in the presence of RDP215 and red in the presence of 9D-RDP215. Data are representative for three independent measurements. A repeat of three heating and cooling scans was performed. The third heating scan was used for graphic representation. For clarity, the DSC curves were displayed on the ordinate by an arbitrary increment. Both peptides exhibit a severe change in the thermotropic phase behavior of the cancer mimics DPPS and POPS, indicating strong membrane destabilization. No effect on the non-cancer mimic DPPC is observed.

Differential scanning calorimetry (DSC) was performed to study potential differences in membrane perturbation and destabilization by RDP215 and 9D-RDP215 (Figure 5). Liposomes composed of the phospholipids DPPS and POPS were used as cancer mimics. Liposomes comprising DPPC were non-cancer representatives. To determine concentration-dependent effects, the respective liposomes were studied in the absence and presence of peptides with lipid-to-peptide molar ratios of 100:1, 50:1 and 25:1, where both peptides were shown to cause severe membrane perturbation of the cancer mimic DPPS (Figure 5; top; left). The phase transition of DPPS in the absence of the peptide exhibits a calorimetric enthalpy (ΔH_m_) of 10.8 kcal/mol, a main transition temperature T_m_ of 52.9 °C and a half-width (T_1/2_) of 0.34 °C (Table 3), which is in accordance to published data [23]. In the presence of RDP215 and 9D-RDP215, this transition peak splits into two or more peaks. Transition peaks at a lower temperature than the main transition temperature T_m_ of pure DPPS can be attributed to so-called peptide-enriched (_pe_) and affected lipid domains, whereas transition peaks at the main transition temperature can be attributed to peptide-poor (_pp_) non-affected domains [23]. At the lowest lipid-to-peptide ratio of 25:1 RDP215 affects the majority of DPPS (75%). The addition of RDP215 results in two transition peaks at a T_m_ decreased by 4.4 °C and 3.9 °C, respectively, implying strong membrane perturbation caused by the peptide. The peptide-affected transition at 48.5 °C is the most prominent peak caused by RDP215 at all three lipid-to-peptide ratios. The ΔH_m pe_ of the peptide-enriched domain at 48.5 °C is increasing with higher peptide ratios, whereas the ΔH_m,pp_ of the peptide-poor domain is decreasing, together with the T_m_ decrease, indicating a strong peptide-induced membrane perturbation and destabilization of the gel phase. Further, the broadening of the transition with an increase in the half-width T_1/2_ up to more than 1 °C confirms this assumption. In addition, RDP215 induces a small transition peak at 39 °C, which, at first sight, might be attributed to a so-called pre-transition from a tilted to a non-tilted gel phase. More likely, the transition, however, might indicate the formation of membrane peptide stacks in the gel phase, which undergo a transition from an ordered gel phase (stacked bilayers with peptide integrated) to a disordered gel phase (single bilayers with peptide integrated) upon the temperature raise. For further verification though, X-ray analyses would have to be performed. This assumption is supported by similar findings induced by N-acylated peptides, wherein X-ray studies exclude a transition from a gel-to-liquid crystalline phase and assign the peak to a transition from an ordered gel to a less ordered gel phase [23]. 

Additionally, 9D-RDP215 induces a splitting of the main transition into peptide-enriched and peptide-poor domains and an additional transition that might be caused by a stack dissolution (already at the lowest peptide lipid ratio 1:100) though to a significantly higher extent. At a lipid-to-peptide ratio of 25:1, this transition even comprises the major proportion of the enthalpy with 3.9 kcal/mol and interestingly high cooperativity of 0.32 °C (Table 3), an attribute that allows excluding a (plain) pre-transition. As in the presence of RDP215 (ΔH_m pp_ 10.3 to 2.4 kcal/mol at T_m_ 52.9 °C), though again to a significantly higher extent, increasing ratios of the D-peptide induce a strong decrease in ΔH_m pp_, the peptide-poor (non-affected) DPPS domain (ΔH_m pp_ 9.5 to 0.2 kcal/mol at T_m_ 52.9 °C), and, in return, increase the peptide-affected PS domains (ΔH_m pe_ 0.6 to 3.5 kcal/mol at T_m_ 50.5 °C–51.3 °C). Thus, the D-peptide induces a much stronger effect leaving at highest peptide ratios only 2% of PS non-affected compared to 25% by RDP215, indicating an even more severe membrane destabilization.

In accordance with the results on DPPS, both peptides also cause severe changes in the phase behavior of the second cancer mimic, POPS (Figure 5; top; right). Pure POPS in the absence of peptides displays a one phase transition with a calorimetric enthalpy (ΔH_m_) of 5.3 kcal/mol, a transition temperature T_m_ of 11.8 °C and a transition half-width (T_1/2_) of 1.76 °C (Table 3), which is consistent with published data [46]. The thermograms of the POPS measurements indicate that both peptides cause a strong reduction in the transition enthalpy ΔH_m_ by about 90% at the highest peptide ratios, implying severe membrane destabilization. In contrast to DPPS, only the addition of RDP215 induces two distinct and visible phase transition peaks comprising a peptide-affected domain at 8.0 °C and a non-affected domain at 11.8 °C. In the case of the POPS thermogram, in the presence of the D-peptide, no peptide-affected domain in the observed temperature range is visible, and the total phase transition enthalpy is decreased to 0.5 kcal/mol. This can be attributed to an even stronger and more uniform membrane destabilization by the D-peptide. Both peptides cause a loss of cooperativity, indicated by the increase in half-width T_1/2_. The DSC studies show that 9D-RDP215 is more effective on the cancer mimics, POPS and DPPS, than the L-peptide.

Contrary to their strong interaction with the cancer-mimics DPPS and POPS, both peptides show no significant effect on the non-cancer mimic DPPC (Figure 5, bottom), suggesting high specificity of both peptides for cancer cells without effecting non-cancerous cells.

As described before [47], DPPC exhibits two characteristic phase transitions. A pre-transition at 35.9 °C with a pre-transition enthalpy of 1.9 kcal/mol and a main transition at 41.6 °C with a transition enthalpy of 10.3 kcal/mol and high cooperativity T_1/2_ of 0.14 °C (Table 3). As depicted in Figure 5 (bottom), the characteristic thermotropic phase behavior of DPPC does not change in the presence of RDP215 or 9D-RDP215 regardless of the lipid-to-peptide ratio.

#### 2.2.4. Membrane Interaction and Penetration by RDP215 and 9D-RDP215

To obtain information on peptide solubility of RDP215 and 9D-RDP215 and their potential penetration depth in PS and PC membranes, tryptophan (Trp) absorbance and quenching studies were performed. Generally, the emission properties of Trp changes with the polarity of its environment and can therefore give information about the localization of Trp in different environments, e.g., buffer or membranes of different composition [23]. First, the emission spectra of RDP215 and 9D-RDP215 were determined in the buffer (PBS) as well as in the presence of liposomes mimicking cancer membranes (DPPS and POPS) and non-cancer membranes (DPPC) to study the solubility or possible aggregation. For comparability, DSC samples with a lipid-to-peptide ratio of 25:1 were also used for the Trp quenching experiment.

**Figure 6:**
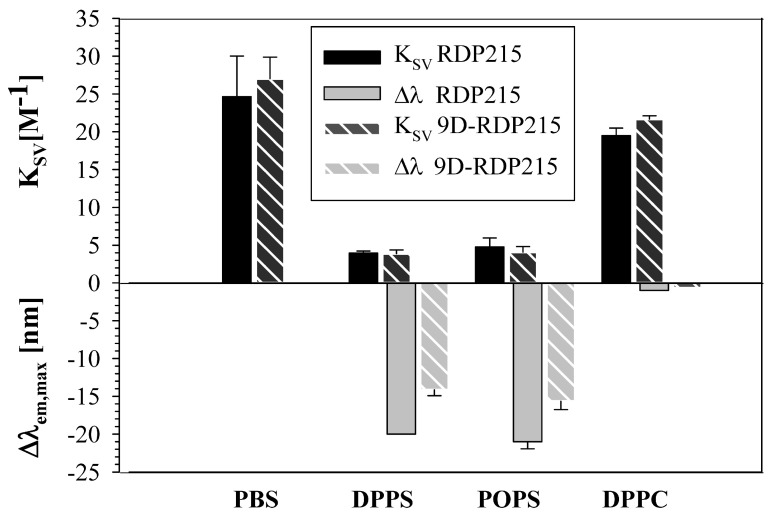
Tryptophan quenching of RDP215 and 9D-RDP215 to determine potential interaction and membrane penetration of the peptides with PS and PC membranes. The Trp quenching was determined in presence of buffer (PBS) or of liposomes composed of DPPS, POPS and DPPC. (DSC samples diluted in PBS buffer (20-400 µL DSC sample in 2 mL buffer; lipid to peptide ratio 25:1) with respective concentrations (0.1 M, 0.4 M and 0.7 M) of acrylamide. The excitation wavelength was 280 nm, and the emission wavelength scans were recorded between 300 nm and 410 nm. The low Stern-Volmer quenching constant (K_SV_), and changes of the maxima of the emission spectrum (Δλ_em, max_,) of both peptides in DPPS and POPS liposomes in comparison to PBS buffer (356 nm) indicate that the Trp of the peptide interacts with and penetrates into both cancer membrane mimics.

In PBS buffer, RDP215 exhibited an emission wavelength maximum (λ_em, max_) of 356 nm with a Stern Volmer constant K_SV_ of 24.7 M^−1^. Moreover, 9D-RDP215 showed a λ_em, max_ at 354 nm with a K_SV_ of 27.0 M^−1^, indicating that the Trp in both peptides are in a polar environment, and therefore, the peptides are completely dissolved and not aggregated in the buffer (Figure 6; Table 4).

In the presence of the cancer mimics, a significant blueshift of the emission wavelength and decrease in the K_SV_ values is induced by both peptides, indicating a strong interaction and penetration of Trp into the hydrophobic membrane interface. In the presence of DPPS liposomes, the emission wavelength maxima of RDP215 exhibits a blue-shift of 20 nm (336 nm) compared to buffer, and the K_SV_ is decreased by 20.7 M^−1^ (4.0 M^−1^) (Table 4). The presence of the second cancer mimic POPS induced a similar effect on the peptides’ Trp. Figure 6 further displays the Trp quenching of RDP215 in the presence of DPPC liposomes. Thereby, no significant alterations in the emission wavelength maximum and K_SV_ values are determined in the presence of the non-cancer mimic DPPC, confirming the specificity of the peptide for cancer-membranes. 9D-RDP215 exhibits similar effects as the L-peptide in the presence of DPPS and POPS as well as DPPC liposomes (Figure 6), indicating sole interaction with the cancer mimic. 

### 2.3. Effect of D-Amino Acids on Peptide Stability-Ex Vitro Studies

The use of peptide-based drugs comprising L-amino acids is particularly limited due to their risk of enzymatic degradation and/or binding to serum components [31,48]. Consequently, the introduction of D-amino acids in peptide therapeutics is considered an enhancement of peptide stability and activity [32]. To investigate such potential differences in the stability of the L-peptide RDP215 and its D-variant 9D-RDP215 in the presence of different sera, the two peptides were incubated for up to 7 days in the absence or presence of 2% and 10% of fetal bovine serum (FBS) and human serum (HS), respectively. 

**Figure 7:**
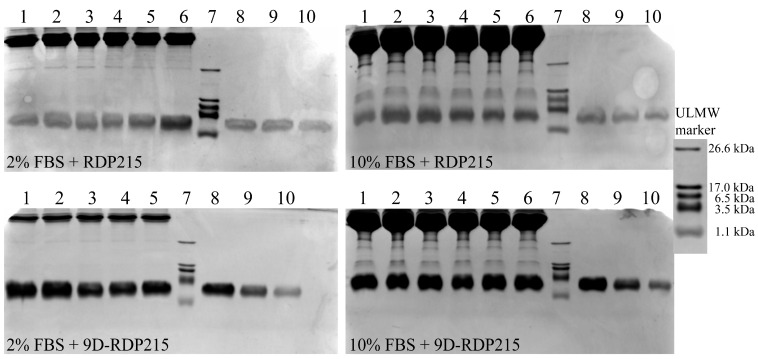
SDS-polyacrylamide gel electrophoresis of stability studies of RDP215 (**top**) and 9D-RDP215 (**bottom**) in the presence of 2% and 10% fetal bovine serum (FBS). Peptides (2.5 µg per sample) were incubated at 37 °C for up to 7 days in the presence of 2% and 10% FBS, respectively. Two percent FBS (**left**) and 10% FBS (**right**): Lane 1–6: 2.5 µg of the respective peptide was incubated in presence of 2% FBS or 10% FBS for 0 h (lane 1), 1 h (lane 2), 8 h (lane 3), 24 h (lane 4), 2 days (lane 5) and 7 days (lane 6). Molecular weights can be estimated from the ULMW marker (lane 7), and fragment sizes are given on the right. Lane 8–10: 2.5 µg, 1 µg and 0.5 µg of the respective peptide control. Bottom left (2% FBS+9D-RDP215), no lane 6 (7 days). Presented data are representative of the results of at least two independent measurements. Neither RDP215 nor 9D-RDP215 show any sign of peptide degradation upon incubation with 2% or 10% FBS.

**Figure 8:**
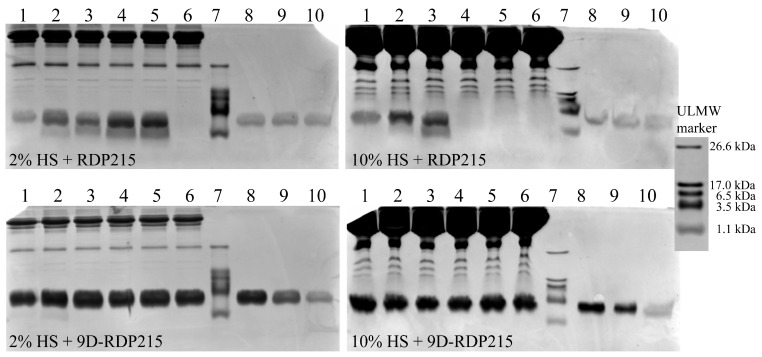
SDS-polyacrylamide gel electrophoresis of stability studies of RDP215 (**top**) and 9D-RDP215 (**bottom**) in the presence of 2% and 10% human serum (HS). Peptides (2.5 µg per sample) were incubated at 37 °C for up to 7 days in the presence of 2% and 10% HS, respectively. Two percent HS (**left**) and 10% HS (**right**): Lane 1–6: 2.5 µg of the respective peptide was incubated in the presence of 2% HS or 10% HS for 0 h (lane 1), 1 h (lane 2), 8 h (lane 3), 24 h (lane 4), 2 days (lane 5) and 7 days (lane 6). Molecular weights can be estimated from the ULMW marker (lane 7), and fragment sizes are given on the right. Lane 8–10: 2.5 µg, 1 µg and 0.5 µg of the respective peptide control. Presented data are representative of the results of at least two independent measurements. While 9D-RDP215 does not show any peptide degradation in the presence of 2% or 10% HS, RDP215 already shows degradation within 7 days (lane 5) of incubation in the presence of 2% and already within 24 h (lane 4) in the presence of 10% HS.

For that purpose, the peptide samples, which were preincubated in the presence of serum, were visualized with Coomassie staining after separation from other serum components as serum proteins such as bovine and human serum albumin on SDS-PAGE (Figure 7 and Figure 8). The comparison with the running pattern of the ULMW marker confirms the peptide molecular weight of 2483 g/mol (2.5 kDa) by appearance between 3.5 kDa and 1.1 kDa. For quantification and control, peptide weight standards (2.5 µg, 1.0 µg and 0.5 µg peptide) were applied to estimate the dimension of potential peptide degradation (initial quantity 2.5 µg). As an additional control, peptides were incubated for up to 7 days in buffer (PBS) without serum (0% FBS/0% HS). No degradation over 7 days was determined for both peptides in buffer (PBS) in absence of serum (Figure S1).

Figure 7 (top) shows the sample aliquots of RDP215 in the presence of 2% and 10% FBS (lanes 1–6). No degradation can be determined after incubation within 7 days (lane 6). Similarly, no degradation of the D-variant 9D-RDP215 in the presence of 2% and 10% FBS was observed (Figure 7; bottom). Due to technical problems in the series of 9D-RDP215 in the presence of 2% FBS, the stability was only studied over 2 days. However, with 10% FBS, it was confirmed that also after 7 days, no degradation took place. Further, in an experimental repeat with less quality in the band pattern, it was shown that there was no degradation of 9D-RDP215 taking place in the presence of 2% of FBS (Figure S2). Thus, no advantage in the presence of FBS regarding proteolytic stability by the introduction of D-amino acids was observed ex vitro. For comparison, a related peptide R-DIM-P-LF11-334, comprised of L-amino acids, already showed degradation in the presence of serum upon incubation exceeding 1 day [25]. In general, the staining of RDP215 appeared weaker than that of the D-peptide. Possible reasons for this effect can be due to the rinsing of L-peptide during electrophoresis or de-staining.

FBS is commonly used for in vitro studies, though for the potential use of peptides as anticancer therapy in humans, the effect of human serum (HS) on peptide stability is even more crucial regarding the drug concentration that is effective on the tumor upon systemic treatment. Thus, Figure 8 displays peptide stability in the presence of 2% and 10% HS. From 1 h to 2 days of incubation with 2% HS, RDP215 shows an increasing amount of additional peptide fragments at a lower molecular weight (down to 1.1 kDa) than the actual peptide weight (2.5 kDa), implying ongoing peptide degradation in the presence of HS (Figure 8; top; lane 2–5). After 7 days, the peptide is already completely degraded, and no peptide (fragment) is visible anymore (lane 6). At 10% HS, RDP215 is already completely degraded upon 24 h (lane 4). On the contrary, the D-peptide shows no degradation in the presence of 2% and 10% HS within 7 days (lane 1–6) (Figure 8, bottom).

In summary, the introduction of D-amino acids is shown to completely restore peptide stability in the presence of human serum. This allows prolonged effective and stable activity of 9D-RDP215 upon treatment, which is of importance for clinical use.

### 2.4. Effect of D-Amino Acids on Peptide Stability-In Vitro Studies in Melanoma

The model studies revealed a slightly increased effect on the cancer mimic by the D-peptide. However, both peptides showed specific interaction solely with PS exposed by cancer cells. In ex vitro studies, an obvious instability of RDP215 in the presence of HS was shown, whereas no effect on stability of the D-peptide was observed in the presence of both serum types of bovine and human origin. In the following in vitro studies, a potential impact on specific and stable antitumor activity is now investigated. To confirm whether the stronger interaction with the PS marker and the higher stability of 9D-RDP215 affect its activity in vitro, the peptide-induced cell death of the melanoma cell line A375 was monitored in the presence of the two different serum types. Since the D-amino acid substitution within the sequences of potent drug candidates should increase their stability to proteolytic enzyme [31,49,50], the effect of protease inhibition on the stability and antitumor activity was additionally examined. Furthermore, the mode of action by which the peptides cause cell death was studied. 

#### 2.4.1. Peptide Toxicity and Specificity for Melanoma A375 and Normal Human Dermal Fibroblasts NHDF under Standard Serum Conditions

To study peptide toxicity and specificity for melanoma, first, peptide-induced cell death was studied under standard culture conditions comprised of 10% FBS. Table 5 lists the LC_50_ values obtained upon 4 h and 8 h of treatment of melanoma A375 with RDP215 and 9D-RDP215, respectively. The LC_50_ values after 4 h (7.0 µM) and 8 h (1.4 µM) reveal that 9D-RDP215 is about 2.5–3 times more efficient in killing melanoma than the L-peptide. This correlates also with increased membrane destabilization of the cancer mimic by the peptide observed in the model studies. Table 5 further lists the LC_50_ values for the peptides affecting the non-neoplastic NHDF cell line. Though also exhibiting increased toxicity on normal cells, 9D-RDP215 still displays a high specificity for cancer cells of 8-fold to 14-fold upon 4 h and 8 h of peptide incubation. This specificity is comparably as high as achieved by the L-peptide; however, a 2- to 3-fold higher antitumor activity can be of great importance in potential clinical use. Further, it has to be considered that the fibroblast growth medium only contains 2% FBS. The highly selective activity of both peptides could also be confirmed with a second melanoma cell line, SBcl-2. Again, the D-peptide already showed higher activity at lower concentrations (Figure S3, Table 5).

#### 2.4.2. Peptide Toxicity and Stability in Presence of Different Concentrations of Fetal Bovine Serum FBS and Human Serum HS

As discussed before, the in vivo use of peptides is severely limited due to the loss of activity in serum, partially because of enzymatic degradation and binding to serum components [48]. The substitution of L-amino acids with their D-form, which are particularly susceptible to enzymatic cleavage, is therefore a strategic approach to prolong the half-life of therapeutic peptides [33,51]. Thus, in the following, the cytotoxicity of RDP215 and 9D-RDP215 was studied in the presence of varying concentrations of FBS and HS to determine the potential impact of different serum type components on the in vitro activity of the respective peptides.

**Figure 9:**
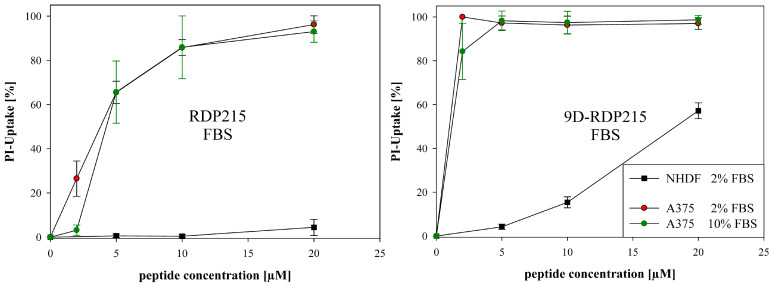
Specific toxicity of RDP215 and 9D-RDP215 on the malignant melanoma cell line A375 and normal human dermal fibroblasts in the presence of 2% and 10% fetal bovine serum (FBS). Cell death (%) was determined by PI-uptake in presence of 2 µM, 5 µM, 10 µM and 20 µM peptide concentration upon 8 h of incubation in the presence of 2% (red circles) and 10% FBS (green circles), respectively. For the determination of cancer specificity, cytotoxicity on normal human dermal fibroblasts NHDF (2% FBS) (black squares) was studied. Both peptides show high activity and specificity for melanoma cells, mainly independent of the concentration of FBS. At lower molar concentration (2–5 µM), the D-peptide exhibits increased stability and significantly increased antitumor activity; however, at higher molar concentrations, cytotoxicity also increases. Data represent mean values of at least 4 independent experiments with standard deviation given.

As already observed in the ex vitro studies, where FBS did not cause any degradation of both peptide types, in vitro studies also revealed no striking effect on peptide activity on melanoma (Figure 9). At lower peptide concentrations (2 µM), however, the L-peptide exhibited a reduced activity in the presence of 10% FBS (Figure 9, green circles) by about 90% after 8 h. The D-peptide, in comparison, was mainly unaffected in the presence of FBS and revealed a significantly increased activity on melanoma already at low concentrations compared to RDP215. The D-peptide also exhibited increased activity on normal human dermal fibroblasts, however, as elevated before, considering the more highly increased antitumor activity, still displays high cancer specificity (see also Table 6).

These results are in good agreement with the ex vitro data, where no peptide degradation upon incubation with FBS was determined for both peptides (Figure 7). The fact that RDP215 shows decreased activity at 2 µM at 10% FBS in vitro might be explained by a higher peptide to serum ratios ex vitro, which correlate to 10 µM of the peptide in vitro. 

**Figure 10:**
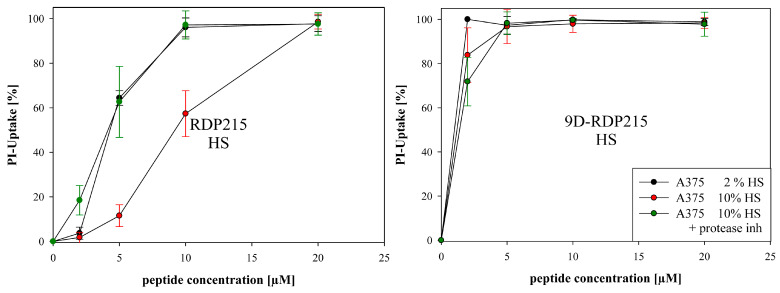
The toxicity of RDP215 and 9D-RDP215 on malignant melanoma cell line A375 in the presence of 2% and 10% human serum (HS). Cell death (%) was determined by PI-uptake in the presence of 2 µM, 5 µM, 10 µM and 20 µM peptide concentration upon 8 h of incubation in the presence of 2% HS (black circles), 10% HS (red circles) and 10% HS with the protease inhibitor (green circles), respectively. The protease inhibitor was added to the cells prior to peptide incubation. Both peptides show high activity for melanoma cells, and the L-peptide exhibits reduced activity in the presence of higher amounts of human serum. In the presence of the protease inhibitor, the activity is restored. Compared, the D-peptide shows increased toxicity on melanoma independent of the concentration of serum. Data represent mean values of at least 3 independent experiments with standard deviation given.

In contrast, ex vitro studies in the presence of human serum (HS) revealed that RDP215 was degraded within 7 days and 24 h at higher serum levels, whereas the D-peptide was not affected (Figure 7). Indeed, also in vitro, 9D-RDP215 was not reduced in its stable activity in the presence of HS, independent of the concentration (Figure 10; right). The L-peptide, on the contrary, showed significantly decreased activity as compared to bovine serum, as well as at higher human serum levels (Figure 10; left). Ex vitro studies were thereby nicely confirmed in vitro.

The results suggest that RDP215 is less stable in HS and consequently also less active than the D-peptide. Due to its high stability in HS and its strong serum-independent activity, 9D-RDP215 is, therefore, more favorable for a potential intratumoral and even systemic application against human tumors in cancer therapy. 

To investigate if the decrease in activity is mainly due to proteolysis, in vitro measurements were expanded by the addition of protease inhibitor to the 10% HS group (Figure 10; green circles). Therefore, the activity of RDP215 at 5 and 10 µM in 2% HS could be restored, revealing proteolytic instability as the main cause for the peptide’s decreased activity in comparison with the D-peptide.

#### 2.4.3. In Vitro Studies on Peptide Induced Killing Mechanism

Two general mechanisms of killing by anticancer peptides are discussed, either triggering necrosis or apoptosis [44]. Necrosis is associated with disruption of the cell membrane, resulting in a release of cytoplasmic content into the surrounding tissue, as well as the induction of inflammation [52]. For therapeutic application, however, “softer” killing by apoptosis exhibiting no release of cellular components, no inflammation and prompt phagocytosis by macrophages or adjacent normal cells, is, therefore, preferred [52]. For related peptides as R-DIM-P-LF11-322 [24] and R-DIM-P-LF11-334 [25], apoptosis was shown to be induced in melanoma cells. 

To determine the mode of action of RDP215 and 9D-RDP215, the RealTime-Glo™ Annexin V Apoptosis and Necrosis Assay was performed. The peptide-induced apoptosis or necrosis signals of malignant melanoma A375 cells were measured every 30 min for 6 h. An increase in PS exposure was detected by binding of Annexin-V, which increases the luminescence signal indicating peptide-induced apoptosis. Necrosis was detected upon peptide-induced loss of membrane integrity, causing an increase in the fluorescence signal. However, it has to be mentioned that the necrosis reagent cannot differentiate between primary and secondary necrosis (late apoptosis) [53]. In in vivo, apoptotic cells would be cleared by phagocytosis, and in in vitro culture, such processes do not occur, which can then lead to late apoptosis and later on secondary necrosis, which is characterized by a disruption of the cell membrane and loss of cell integrity [53].

**Figure 11:**
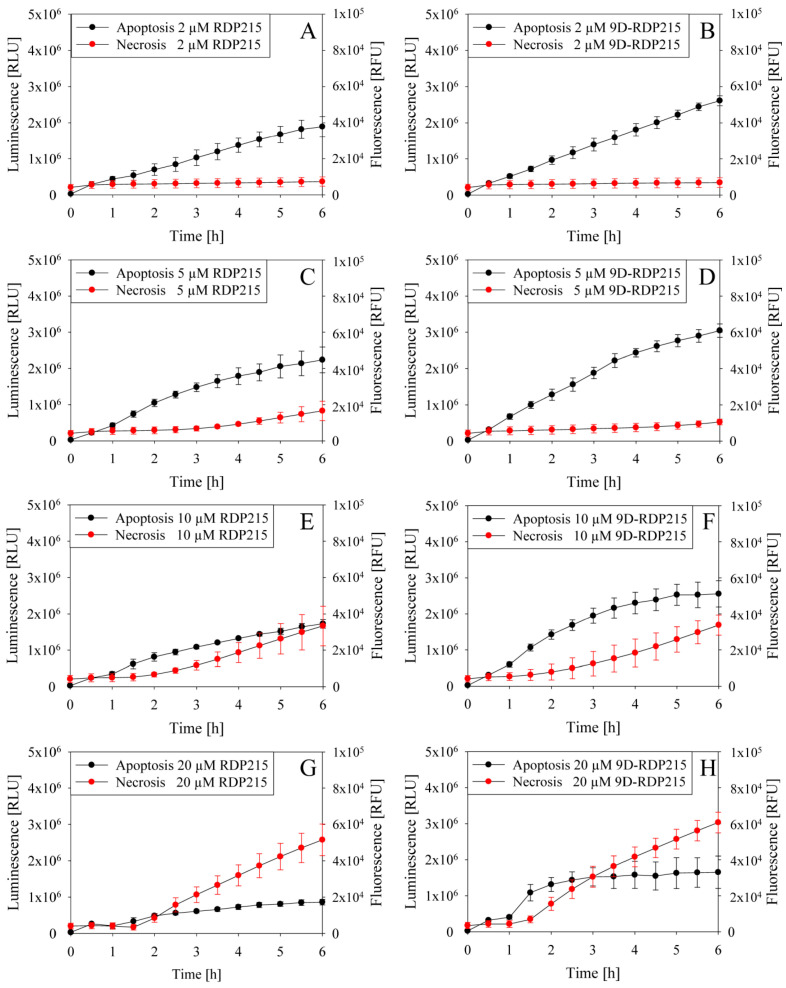
A study of RDP215 and 9D-RDP215-induced cell death in melanoma A375 by apoptosis or necrosis. (**A**) RDP215 2 µM; (**B**) 9D-RDP215 2 µM; (**C**) RDP215 5µM; (**D**) 9D-RDP215 5 µM; (**E**) RDP215 10 µM; (**F**) 9D-RDP215 10 µM; (**G**) RDP215 20 µM; (**H**) 9D-RDP215 20 µM. Apoptosis (black; left scale luminescence) and necrosis (red; right scale fluorescence) signals were measured every 30 min for 6 h. Background subtracted signals are depicted. The mean values with standard deviations are derived from 4 independent determinations. Both peptides kill melanoma cells by induction of apoptosis. However, the D-peptide causes higher levels of apoptosis and, therefore, a higher cell death rate.

As seen in Figure 11, both peptides induce apoptosis in melanoma, the D-peptide, however, to a higher extent, which might correlate to an increased killing efficiency observed in vitro. At higher peptide concentrations (10–20 µM), in addition, killing by necrosis, or presumably late apoptosis, occurs starting at 1–2 h of peptide incubation. So, both peptides exhibit their LC_50_ (3.8 to 1.4 µM) at peptide concentrations that cause a soft killing of melanoma cells by apoptosis.

To discriminate between early and late apoptosis (secondary necrosis), induction of caspase 3/7 activity was assessed. Thereby, it was shown that upon four hours of incubation, 10 and 20 µM of the L-peptide increase intrinsic and/or extrinsic (early) apoptosis by 1.6-fold to 1.9-fold. The D-peptide, however, only showed slight induction of early apoptosis by 1.2-fold (Figure S4). This indicates a stronger impact on killing by late apoptosis and/or primary necrosis by the latter.

### 2.5. Effect of D-Amino Acids on Peptide Toxicity—2D In Vitro Studies in Glioblastoma

PS exposure as a potential target for cationic antitumor peptides has been shown for several cancer types [28]. Nevertheless, in our studies, we tried to focus on cancer with poor outcomes and prognosis, such as malignant melanoma and glioblastoma. In the following, the stable and specific toxicity of D-amino acids is analyzed on the glioblastoma cell line LN-229.

#### 2.5.1. Peptide Toxicity and Specificity for Glioblastoma LN-229 and Normal Human Dermal Fibroblasts NHDF

2D PI-uptake studies were performed over 8 h to assess the specificity and toxic activity of the peptides RDP215 and 9D-RDP215 against glioblastoma cell line LN-229 and fibroblasts NHDF.

**Figure 12:**
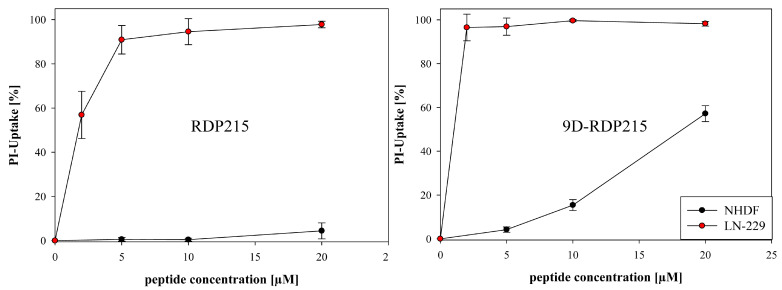
The cytotoxicity of RDP215 and 9D-RDP215 on glioblastoma cells LN-229 and healthy cells NHDF. Cell death was determined by PI-uptake (%) in the presence of 2 µM, 5 µM, 10 µM and 20 µM peptide concentration after 8 h of incubation. With LN-229, RDP215 already displays high peptide-induced cell death up to 90% at 5 µM. Within the studied concentration range, RDP215 exhibits no harm on NHDF cells. 9D-RDP215 shows increased antitumor and non-tumor toxicity, though still with high specificity.

With the glioblastoma cell line LN-229, at low concentrations of 2 µM, RDP215 revealed about 60% and at 5 µM up to 90% of peptide-induced cell death (Figure 12), indicating a high activity at already low concentrations. An LC_50_ of 1.7 µM and minimal toxicity on the non-cancer cell line NHDF up to 20 µM revealed a high specificity for the glioblastoma cells of about 40-fold (Table 6), which is even twice the specificity observed for melanoma (Table 5).

9D-RDP215 exhibits comparable specificity and LC_50_ levels on both cancer types (LC_50_ A375 and LC_50_ LN-229 of 1.4 µM and about 14-fold specificity for the cancer cell line, see Table 5 and Table 6). Due to the fact that the introduction of the D-amino acid not only increases the toxicity on the cancer but also on non-cancer cells, it exhibits lower specificity on glioblastoma than the L-peptide. Nevertheless, at low concentrations, its specificity exceeds the one of the L-peptide. However, it must be mentioned that the L-peptide seems to be more effective on glioblastoma than on melanoma, the reason though is not known. Both peptides were also highly active for a second glioblastoma cell line, U-87 mg. Again, the D-peptide already showed significantly increased activity at lower concentrations (Figure S5, Table 6).

#### 2.5.2. Studies on Peptide Induced Killing Mechanism

**Figure 13:**
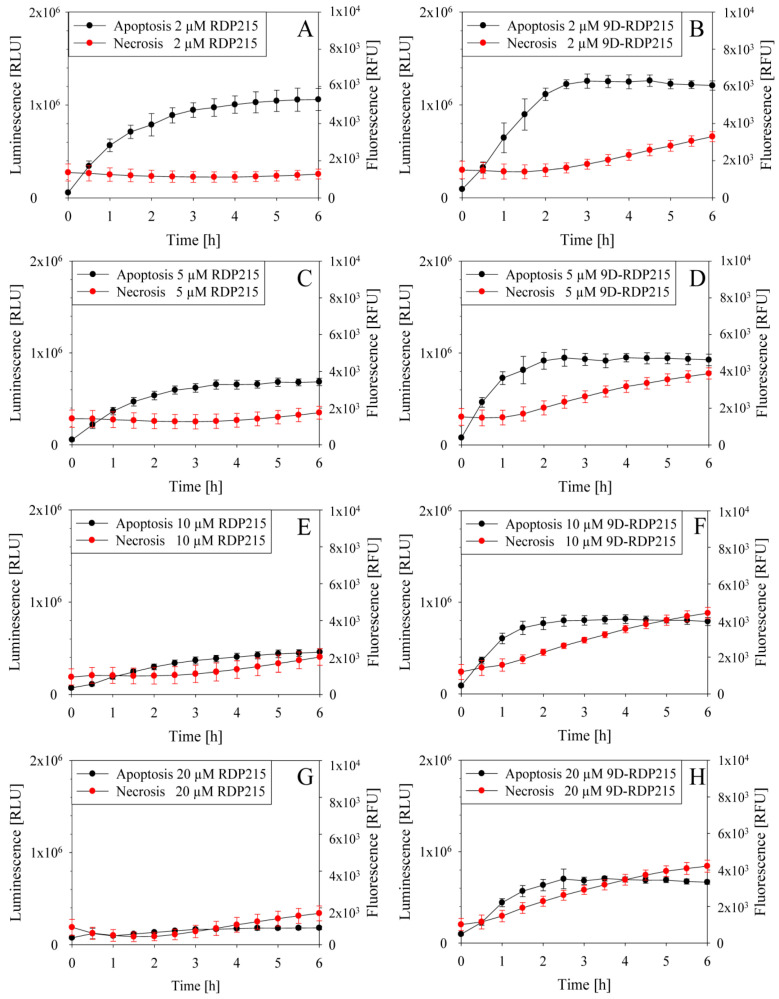
Study of RDP215 and 9D-RDP215-induced cell death in glioblastoma LN-229 by apoptosis or necrosis. (**A**) RDP215 2 µM; (**B**) 9D-RDP215 2 µM; (**C**) RDP215 5µM; (**D**) 9D-RDP215 5 µM; (**E**) RDP215 10 µM; (**F**) 9D-RDP215 10 µM; (**G**) RDP215 20 µM; (**H**) 9D-RDP215 20 µM. Apoptosis (black; left scale luminescence) and necrosis (red; right scale fluorescence) signals were measured every 30 min for 6 h. Background subtracted signals are depicted. The mean values with standard deviations are derived from 4 independent determinations. Both peptides kill glioblastoma cells by the induction of apoptosis. However, overall, the D-peptide causes higher levels of apoptosis and, therefore, a higher cell death rate. Further, necrosis is also minorly responsible for cell death at all concentrations of 9D-RDP215 and increases with rising peptide concentration, whereas apoptosis overall decreases.

As for melanoma and glioblastoma, the mode of action by which the peptides cause cell death was studied. In Figure 13, apoptosis (luminescence, black) and necrosis (fluorescence, red) induction in glioblastoma cell line LN-229 are depicted upon treatment with increasing amounts of RDP215 (left) or 9D-RDP215 (right), respectively. Both peptides kill glioblastoma cells via the induction of apoptosis, whereas, as with melanoma, the D-peptide also shows higher induction of this type of cell death. At higher peptide concentrations, the L-peptide starts to induce slight levels of secondary necrosis or late apoptosis indicated by a time-shift of about 2 h of the fluorescence increase. The D-peptide already induces necrosis/late apoptosis at lower concentrations. 

To discriminate between early and late apoptosis (secondary necrosis), the induction of caspase 3/7 activity was assessed. Thereby, it was shown that upon two hours of incubation, 5 and 10 µM of the L-peptide increase intrinsic and/or extrinsic (early) apoptosis by 1.4-fold to 1.5-fold. The D-peptide did not show significant induction of early apoptosis (Figure S4). This indicates a major impact on killing by late apoptosis and/or necrosis by the latter.

### 2.6. Effect of D-Amino Acids on Peptide Toxicity—3D In Vitro Studies on Glioblastoma Spheroids

Compared with traditional 2D cell culture, 3D spheroidal cell aggregates (e.g., spheroids) are regarded as more physiological and have found application in the field of oncology [54]. Spheroids more properly mimic the tissue-like properties of tumors necessary for the evaluation of compounds of interest, whereas cells in monolayer culture lose many of their in vivo characteristics, which results in a limited predictive value in determining the clinical outcome [55]. Therefore, 3D multicellular tumor spheroids (MCTS) were generated using glioblastoma cell line LN-229 to investigate the toxicity of RDP215 and 9D-RDP215 under improved physiological conditions. 

#### 2.6.1. Direct Toxicity-PI-Uptake of MCTS and Concentration Dependence of Killing by RDP215 and 9D-RDP215

**Figure 14:**
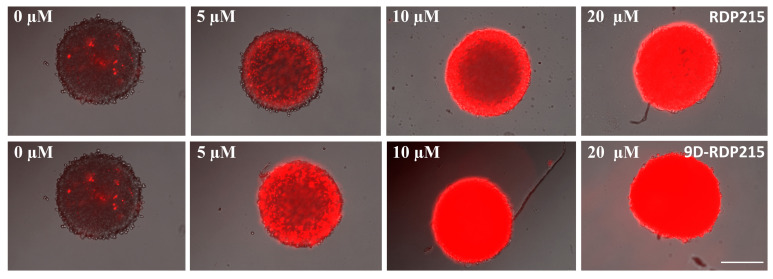
The effect of RDP215 (**top**) and 9D-RDP215 (**bottom**) on LN-229 multicellular tumor spheroids (MCTS) upon 24 h of peptide incubation. Red fluorescence of glioblastoma spheroids by propidium iodide indicates peptide-induced cell death. According to the monolayer experiments, RDP215 causes already high levels of peptide-induced cell death at low concentrations (5 µm), and its activity rises with increasing peptide concentration. The introduction of D-amino acids within 9D-RDP215 significantly increases peptide toxicity on spheroids of LN-229. Microscopic pictures represent data of at least three independent experiments. The scale bar indicates 250 µm.

For fluorescence microscopy studies, MCTS of LN-229 were grown for 5 days and consequently treated with respective peptides at concentrations of 5 µM to 20 µM for 24 h. Then, the extent of cell death in the tumor spheroids was visualized by fluorescence microscopy (Figure 14). 

PI-uptake (red staining) induced by RDP215 revealed a comparable effect on LN-229 MTCS as in the 2D experiments. Already, at low concentrations, RDP215 causes high levels of peptide-induced cell death, indicated by a strong red fluorescence signal. Cell death increases with the rising peptide concentration. MCTS without peptide treatment served as a negative control, monitoring the extent of cell death in the absence of a peptide. RDP215 exhibits a significantly strong cytotoxic effect compared to the untreated control.

In agreement with the 2D results, 9D-RDP215 (Figure 14; bottom) also shows increased toxicity on MCTS of glioblastoma compared to the L-peptide. The tumor spheroids show induction of 100% cell death at 5 to 10 µM D-peptide, whereas the L-peptide does not reach 100% killing before 10 to 20 µM. Naturally, 3D toxicity needs higher peptide amounts than 2D toxicity, due to increased cell-peptide ratios and necessity for peptides of penetration into the spheroid.

#### 2.6.2. Effect of D-Amino Acids on Impairment of Viability of MCTS upon Passage through Blood Brain Barrier

The blood-brain barrier (BBB) is centrally positioned within the neurovascular unit (NVU) and is formed by a tightly sealed monolayer of endothelial cells [56]. The tight junctions of the NVU between the endothelial cells are responsible for the barrier function of the BBB [57]. Various drugs are discovered to treat central nervous system (CNS) disorders but fail to enter the market because of their inability to cross the BBB [19]. 

**Figure 15:**
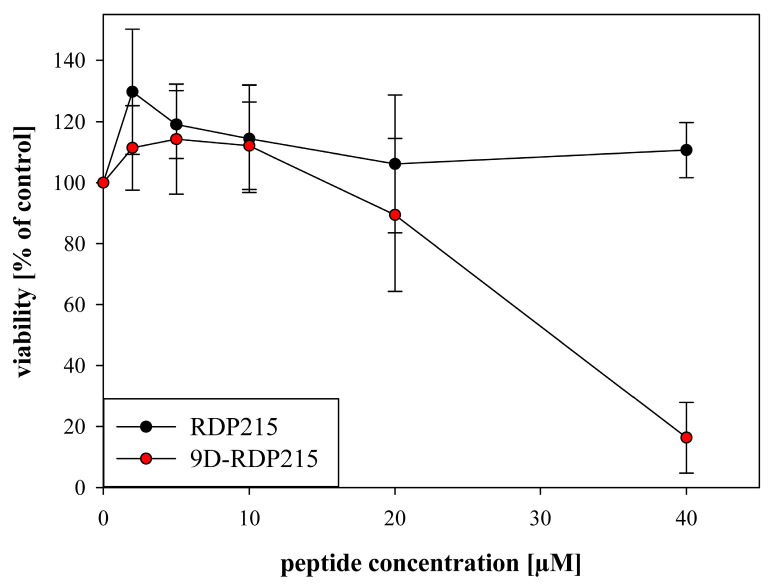
3D cell viability of glioblastoma LN-229 spheroids upon passage through a blood-brain barrier model (MDCKII (hMDR1 KI) monolayer) in dependence of RDP215 and 9D-RDP215. A blood-brain barrier (BBB) model was used to investigate the viability of glioblastoma MCTS upon 48 h of peptide incubation from 2–40 µM. Untreated glioblastoma spheroids were used as negative control, representing 100% of viable cells. The 3D assay reagent measures ATP (adenosine triphosphate) as an indicator of viability and generates a detectable luminescent readout. At the respective concentrations, only the D-peptide was able to pass the epithelial monolayer and to reveal significant and high cytotoxic effects on the LN-229 spheroids. Data points represent mean values with respective SD of at least three independent experiments.

To study the potential of peptide RDP215 and the potential impact of D-amino acids to pass the BBB and consequently exert effective antitumor activity, a three-dimensional glioma BBB model [58] was used. The BBB was simulated using MDCKII (hMDR1 KI) cells as epithelial monolayers. These cells were seeded into Corning^®^ HTS Transwells onto permeable support of a 0.4 µm PET membrane, recreating the BBB. The LN-229 cells again were used as MCTS and were grown separately. After 5 days, the epithelial monolayer was placed above the glioblastoma spheroids, and the permeability and cytotoxicity of the peptides RDP215 and 9D-RDP215 on glioblastoma spheroids was investigated at concentrations from 2–40 µM. After 48 h of peptide incubation, the potential impact on the viability of the glioblastoma (LN-229) spheroids after passage through the BBB was analyzed (Figure 15). In addition, after each viability assay, the monolayer integrity was tested with lucifer yellow (LY), and wells with LY permeability > 5% were rejected for analysis due to damage to the barrier model. Within the studied concentration range, only the D-peptide was able to pass the BBB and consequently exert significant antitumor activity on the glioblastoma spheroids. At 40 µM, the D-peptide is already able to induce about 80% of cell death of the 3D tumor model.

## 3. Discussion

Small peptides, as derived from the human host defense peptide lactoferricin (hLcin), are a promising novel strategy in cancer therapy [22,28]. Because of their cationic and amphipathic properties, the peptides interact specifically with negatively charged membranes via electrostatic and hydrophobic interactions [28]. In 2011, Riedl et al. were able to confirm reports that cancer cells expose the negatively charged lipid phosphatidylserine (PS) in the outer leaflet of their plasma membrane, representing a promising target for cationic host defense peptides [27,43,59], in contrast to the cell surface of non-cancer cells that expose neutral (zwitterionic) components, such as phosphatidylcholine (PC) [42,60]. Neutral cell membranes are not affected by the peptides, and thus, the peptides do not enter or harm non-malignant cells. Such specificity for cancer membranes enables a reduction of side effects in a potential therapy [28].

Consequently, optimization in the design of human lactoferricin derivatives is of high interest to develop effective antitumor therapeutics with decreased side effects. A common strategy to improve peptide-based drugs regarding their selectivity and stability is the substitution of L-amino acids with their D-enantiomers [35,61]. Therefore, the aim of this study is to investigate the potential influence of D-amino acids on the design of antitumor peptides regarding stability, activity and selectivity and thereby following a potential approach towards peptide optimization. The focus was on the two human lactoferricin derivatives: RDP215 (R-DIM-P-LF11-215) [26] and 9D-RDP215. They share the same amino acid sequence, but within 9D-RDP215, several amino acids are replaced by their D-form at potential proteolytic cleavage sites (Arg and N-and C-terminal Phe). In general, the peptide RDP215 exhibits a two β-strand structure separated by a loop introduced by a proline in the middle of the sequence (PEP-fold structure prediction). Notably, many studies report the necessity of membrane-active peptides forming a linear α-helical structure; however, only 2% of known antitumor peptides exhibit a β-sheet structure [28]. However, in previous studies, Riedl et al. showed that mainly LFcin derivatives exhibiting a looped structure with 2 β-strands or 2 α-helices were able to act specifically on cancer cells without the exhibition of any cytotoxicity on normal cells [24]. Further, in the present study, CD-spectroscopy data reveal that the L- and the D-peptide only increase their ß-sheet conformation in the presence of the cancer target PS, whereas in the presence of the non-cancer mimic PC, the structure resembles the one in the presence of buffer, promising cancer selectivity in vivo. This is in perfect agreement with data on related and cancer-specific peptides R-DIM-P-LF11-322 [23,24] and R-DIM-P-LF11-334 [25], where it was shown that for the formation of a membrane active structure of peptides, interaction with PS exposed by cancer cells is needed. In buffer, as well as in a neutral environment, such as non-cancer plasma membranes, selective peptides remained mainly unstructured. In fact, in the presence of buffer and PC, RDP215 was mainly unstructured; however, the D-peptide already exhibited a higher proportion of the ß-sheet structure in the buffer. This probably causes a stronger interaction with membranes, which, in general, partially explains the increased toxicity on cancer and non-cancer cells compared to its L-analog. Additionally, the linear α-helical peptide DIM-LF11-318, showing increased but non-selective activity, already exhibited a high proportion of the secondary structure in the solution [24]. However, contrary to 9D-RDP215, the structure changed in the presence of non-cancer and cancer mimics [24], which might cause its low selectivity. Further, according to the secondary structure prediction, 9D-RDP215 comprises a higher number of hydrogen bridges, which, in addition to the reduced accessibility for proteases, might cause an increase in stability and consequently in activity.

Besides CD, other biophysical studies were performed to investigate the selective interaction of the two peptides with cancer cells. Therefore, liposomes mimicking cancer (PS) and non-cancer (PC) membranes were generated, and membrane permeability (leakage studies), perturbation and destabilization (DSC) and penetration (tryptophan quenching) were shown to be induced by the peptides only when the cancer marker PS was present. No effects of the peptides on the non-cancer marker PC were observed. The membrane permeability studies further revealed that a certain concentration of peptides is needed to cause sufficient leakage of POPS liposomes, suggesting that the peptides interact with the liposomes according to mechanisms, as proposed by the “carpet” [44] or the “sinking raft” model [62]. Cationic peptides first associate with negatively charged molecules at the surface of membranes, thereby covering it in a “carpet-like” manner [44]. When a certain threshold of peptide concentration is reached, the peptides then insert into the membrane, causing membrane permeabilization [44]. Alternatively, upon attraction to target molecules on the cell membrane, they sink through the bilayer into the cell [62] to reach an inner target, such as, for example, the Golgi or mitochondria, inducing stress and the start of apoptosis [24,63,64]. For related peptides, it had already been excluded that uptake occurs via clathrin or caveolae-mediated endocytosis [64]. The mechanism by which the peptides permeate liposomes differs from that exerted by highly lytic but also often unspecific peptides, such as melittin, which already induces leakage at low concentrations [44]. In the following, the DSC experiments also confirmed the cancer-specificity of both peptides. RDP215 and 9D-RDP215 interact with PS membranes (DPPS and POPS), causing changes in the characteristic phase transition behavior of the negatively charged phospholipid. RDP215 and 9D-RDP215 induced severe PS membrane perturbation and destabilization, suggesting that both peptides strongly interact with the cancer marker PS. Compared to RDP215, the effect of the D-peptide on PS membranes was even stronger regarding the more highly decreased enthalpy and cooperativity of the treated DPPS and POPS liposomes, which might, at least partially, correlate to higher activity in vitro, as shown for the related peptide R-DIM-P-LF11-322 compared to the short peptide comprising only one peptide moiety (LF11-322) [23,25,26]. The Trp-quenching studies show that if the peptide acts specifically against a certain membrane lipid, e.g., PS, its Trp exhibits a significant blue-shift of the emission wavelength upon interaction with the membrane lipid [24]. The blue-shift indicates that the Trp is located in a more hydrophobic environment within the membrane due to the interaction with the membrane interface [24,47]. RDP215 and 9D-RDP215 both exhibited a blue-shift in the presence of the cancer-mimic PS, whereas in the presence of PC liposomes, no blue shift was determined. The absence of such a blue-shift indicates that the peptides do not enter the non-cancer membrane, confirming the specific interaction with cancer membranes.

Differences in the interaction of both peptides in the presence of PS membranes might be associated with the contained amino acid enantiomers, as described by Li et al. [32]. In their study, it is reported that at a conceptual level, host defense peptides comprising D-amino acids recognize their targets in an identical way as those with all L-amino acids [32]. The first step of recognition and binding to the target, negatively charged molecules, at the target membrane is accomplished by electrostatic attraction by both types of cationic peptides [65]. Indeed, the liposomal model studies confirmed that both peptides interact with their target, PS, suggesting that both peptides are able to recognize and bind to PS membranes. Additionally, for the related L-peptide R-DIM-P-LF11-322, it was seen that enzymatic conversion of the cancer cell exposed PS to phosphatidylethanolamine (PE) correlated with decreased peptide activity on melanoma cells [64], indicating a first interaction point with the target lipid PS. Within bacterial membranes, L-peptides were observed to insert into the outer membrane of the lipid bilayer, whereas their D-enantiomers were only surface localized [35,66]. Once the L-peptide is bound to the membrane surface, hydrophobic interactions enable it to further penetrate into the hydrophobic core of the membrane [35]. It is suggested that the activity of D-peptides almost predominantly depends on electrostatic interactions, whereas L-peptides are also capable of a considerable level of hydrophobic interaction for the binding and subsequent permeation into zwitterionic membranes [32,65]. Indeed, in our studies, the penetration depth within PS membranes as well as leakage thereof was slightly decreased or decelerated by the presence of D-amino acids. However, more strongly decreased phase transition enthalpy of PS liposomes (DSC) and the increased structure in the buffer suggest higher hydrophobicity exerted by the D-peptide, which contrasts with reports about lower hydrophobicity exerted by D-peptides.

Following the model studies, potential differences in in vitro activity were investigated with melanoma and glioblastoma. In general, the use of L-amino acid peptides in vivo is particularly limited because of their risk of enzymatic degradation and binding to serum components [48]. Peptide-based drugs applied systemically enter the bloodstream, where they are confronted by proteases and other factors engaged in processes of high importance in the case of injury, and in particular, wound healing [31]. Thus, a major limitation of peptide pharmacokinetics is their short half-life in the bloodstream due to proteolytic cleavage by proteases and peptidases [67]. One of the main enzymes in peptide degradation, dipeptidyl peptidase IV, is not the major challenge for the peptides within this study since both lack its preferential N-terminal cleavage sites Pro1 or Ala1 [61]. Though, calcium- and vitamin K-dependent serine proteases required in blood coagulation cause trypsin-like proteolysis with cleavage sites C-terminal to Lys or Arg residues in the P1 position of particular sequence motifs [31], enabling a potential degradation of therapeutic peptides comprising respective cleavage sites [49]. This is considered in the design of 9D-RDP215 by the substitution of D-amino acid residues at respective potential cleavage sites of RDP215. 

To determine serum-dependent effects on the stability and, consequently, on their activity, the peptides were incubated with FBS and HS. For these studies, commercially purchased sera, FBS (Gibco^®^, Thermo Fisher Scientific) and HS (Sigma-Aldrich), were used. In general, serum is similar in its composition to plasma, excluding factors involved in blood coagulation, such as fibrinogen [68]. The most abundant plasma/serum proteins are albumin and globulins [68]. Heger et al. demonstrated that the albumin concentration in HS is higher than in FBS, which could influence the stability and, consequently, the activity [69]. Besides the concentration differences, species-specificity of certain factors might also have influence on the applied sera [69]. The related peptide R-DIM-P-LF11-334 (lack of F1 and F17) showed 75% binding to BSA upon the seventh day, though without any degradation. Nevertheless, these binding studies are difficult to interpret, and FBS itself and other components induced complete degradation after more than one day of incubation. Still, killing of melanoma, which appeared within 8 h, was complete within this time period [25]. The ex vitro stability studies for RDP215 now revealed that upon incubation in FBS at the respective concentrations and ratios, both peptides, L- and D-form, were stable. However, RDP215 was shown to be degradable upon incubation with HS, while there was no effect on 9D-RDP215, confirming that D-amino acid residues in the peptide sequence indeed increase the stability [49]. That degradation of RDP215 in HS was due to proteolysis, which was further confirmed using the respective inhibition of proteolysis that re-established the activity of the L-peptide. Such a susceptibility to increased degradation in HS compared to FBS was also shown by related L-peptides R-DIM-P-LF11-334, R-DIM-P-LF11-322 and others [70]. Considering the potential application of the peptides as cancer therapeutics in humans, the stabilization by D-enantiomers, as shown by 9D-RDP215 in HS, is of great requirement and sounds promising.

Upon standard conditions (10% FBS), the LC_50_ values of the peptides for melanoma revealed that a 2.5-times lower peptide concentration of the D-peptide is required to result in the same killing efficacy as RDP215. Further, the LC_50_ value of 9D-RDP215 after 8 h of incubation is below 2 µM. These results already reveal that the D-peptide has a higher activity against cancer cells and that this increased killing efficiency is already evident at low peptide concentrations. This is associated with the higher stability of the D-peptide (ex vitro) and the stronger membrane destabilization of PS (DSC). Besides, the determined LC_50_ values of the peptides against non-neoplastic normal dermal human fibroblasts (NHDF) show a comparablly high specificity for cancer cells for both peptides of about 15-fold upon 8 h of incubation, confirming the liposomal model studies that suggested no effect of the peptides on healthy cells at concentrations needed for sufficient killing of cancer cells.

One major concern in using non-natural D-amino acids is the potential toxicity [30]. Indeed, 9D-RDP215 shows a higher toxicity against non-neoplastic cells than RDP215. However, the advantage of 9D-RDP215 is that already very low amounts of the D-peptide are sufficient for a high killing efficacy of melanoma and glioblastoma cells. The application of such small amounts of peptide makes the D-peptide favorable for clinical use over RDP215.

The cytotoxicity of the peptides was further studied in the presence of different amounts of FBS and HS to observe the effect of the serum concentration and components not only on the stability (ex vitro studies) but also on the activity of the peptides. The studies revealed no significant serum concentration or type dependence for the D-peptide, confirming its higher killing efficacy. The results further revealed the instability of RDP215 in HS and associated lower activity in in vitro experiments in the respective serum, which supports the use of the D-peptide in therapy of human tumors.

Further, in vitro toxicity studies on glioblastoma cell line LN-229 revealed the antitumor activity of both peptides in two-dimensional as well as in three-dimensional culture in accordance with the promising liposomal model system studies. Therefore, the ability of both peptides to target and interact with negatively charged PS, which is, as in melanoma, also specifically exposed on glioblastoma cells [27], was confirmed in vitro as well. Like in liposomal studies, 9D-RDP215 displayed higher cytotoxicity levels on glioblastoma cells in comparison to RDP215, therefore confirming higher efficiency probably due to conformational changes by D-amino acids. This might be associated with the increased stability of the D-peptide against proteolytic degradation in the presence of 10% FBS used for the cell culture studies. Both peptides showed high activity already at low concentrations, resulting in a calculated lethal peptide concentration at which 50% of the cancer cells are killed (LC_50_) in the presence of less than 2 µM peptide, indicating potential good therapeutically applicability for both peptides in the treatment of glioblastoma. Regarding specificity comparison with the LC_50_ values for NHDF yields a forty times higher selectivity of RDP215 for glioblastoma cells LN-229, proposing low side effects on healthy cells in cancer treatment in case of this therapeutic peptide. The D-peptide showed an even stronger cytotoxic effect on glioblastoma LN-229 cells, though, in contrast to RDP215, 9D-RDP215 is only ten times more likely to kill glioblastoma cells than healthy cells. According to its high antitumor activity, however, and due to the fact that at 1.4 µM, the D-peptide already kills 50% of the glioblastoma LN-229 cells, whereas healthy cells are only noteworthy harmed at peptide concentrations below 10 µM, the D-peptide still could be preferably used due to its high activity, specificity and stability at very low concentrations. 

Regarding the specific killing mechanism, two general patterns exerted by anticancer peptides are discussed, either triggering necrosis or apoptosis [24,28]. The first is a rapid killing mechanism is proposed for triggering necrosis; thereby, the peptides directly kill cancer cells by disrupting the target plasma membrane [44]. A slower and more controlled and therefore favorable killing of cancer cells combined with membrane blebbing and increased PS exposure indicates membrane-mediated apoptosis [24,28,44]. For triggering apoptosis, the related peptide R-DIM-P-LF11-322 was shown to first enter the cell specifically via PS exposing sites on the surface of cancer cells to further reach negatively charged targets on the surface of mitochondria and/or Golgi, such as PS, phosphatidyl inositol or cardiolipin [24,44]. Riedl et al. were able to prove this association by caspase-3/7 cleavage studies as well as via apoptotic DNA fragmentation analysis [24], studies with PS depletion on cancer cell plasma membranes, induction of mitochondrial swelling and vesicle trafficking upon specific peptide entrance in melanoma cells upon localization to Golgi [24,64].

The mode of action within this in vitro study reveals that both peptides (D- and L-) cause cell death in melanoma as in glioblastoma via the induction of apoptosis rather than (primary) necrosis, which is favorable for potential clinical use due to the prevention of inflammatory processes upon the treatment of cancer patients. The D-peptide even caused increased levels of apoptosis and, even at lower concentrations, presumably late apoptosis or primary necrosis compared to the L-peptide. The time lag of 1–2 h in the onset more likely suggests the induction of late apoptosis. Furthermore, it has to be considered that in vivo apoptotic cells are cleared by immune cells that are not present in vitro. Therefore, at a certain point in apoptosis, the degradation of DNA and membrane disintegration occurs. In general, apoptosis is blocked in many cancer types as in melanoma cells by the inhibition of a gene encoding the apoptotic protease activating factor-1, Apaf-1 [29]. The progression of melanoma and its survival is dependent on the manipulation of cell death pathways, the escape from the immune surveillance and the survival in an unfavorable microenvironment [71]. Additionally, glioblastoma is known to be highly resistant to apoptosis and only shows moderate autophagic cell death, which is partly the reason for a poor therapeutic response to conventional therapies [72]. Therefore, the development of therapies that can restore apoptosis in melanoma and glioblastoma is of great interest in cancer research.

Furthermore, 3D studies were conducted, creating glioblastoma spheroidal cell aggregates (e.g., spheroids). Spheroids mimic the tissue-like properties of tumors necessary for the proper evaluation of compounds of interest, whereas cells in monolayer culture lose many of their in vivo characteristics, which results in a limited predictive value in determining the clinical outcome [55]. Both peptides were also shown to exhibit their high antitumor activity on multicellular tumor spheroids, but understandably higher concentrations were needed to exert the same cytotoxic effect as in monolayers. This seems reasonable since it is more difficult for the peptides to penetrate into the core of multicellular cell aggregates and exert their full activity thereon than on cell monolayers. In comparison, 9D-RDP215 showed stronger cytotoxic effects in the 3D experiments in contrast to RDP215. This might be based on the previously proposed mechanism of the L-peptide having a stronger hydrophobic effect and therefore penetrating deeper into the membrane and the D-peptide being localized on the surface [32,35,66]. Accordingly, the D-peptide could be in contact with the neighboring cell and therefore be able to penetrate deeper into the spheroids at even lower concentrations. Moreover, the increased stability at higher serum levels makes the D-peptide more preferentially useful over RDP215 due to its overall higher activity, also leading to a higher penetration and killing of tumor spheroids at low concentrations. 

Not only resistance to treatment is a problem for therapy of malignancies in and to the brain, passage through the blood brain barrier (BBB) is also a hindrance for most of the standard therapies [18].

The blood-brain barrier (BBB) is formed by a tightly sealed monolayer of endothelial cells [56], and the tight junctions between the endothelial cells are responsible for the barrier function of the BBB. Various drugs are discovered to treat central nervous system (CNS) disorders but fail to enter the market because of their inability to cross the BBB [19]. Vemurafenib, an antitumoral agent that is capable to pass the BBB, was approved in 2011. It inhibits the BRAF-serine/threonine-kinase and leads to a median survival of 13.6 months compared to 9.7 months upon treatment with dacarbazine [73]. Nevertheless, vemurafenib causes severe side effects [74,75] and depends on a special mutation in BRAF, and therefore, applicability is limited.

For small water-soluble cationic, amphipathic and lipophilic peptides, passage through the BBB can be possible. For example, peptides, such as RVG29 [20] or apamin, can cross the barrier and can therefore be used as so-called Trojan horses to shuttle other (larger) compounds to the brain. In the case of antitumor peptides, they might even be able to embody the shuttle and the cancer-specific invader all in one without toxic effects on normal cells [24,25,26,64]. In the present study, the capability of RDP215 and 9D-RDP215 for permeabilization of the BBB and cytotoxicity on glioblastoma spheroids was tested with a three-dimensional glioma BBB model [58]. Both peptides were able to pass the endothelial monolayer; however, only the D-peptide consequently revealed significant cytotoxic effects on the glioblastoma multicellular cell aggregates. At 40 µM, more than 80% of the tumor cells were killed. The higher activity of 9D-RDP215 might result from conformational changes leading to a higher structure in the solution and higher stability against degradation and therefore longer circulation half-time [34], in contrast to RDP215, which cannot reach the tumor spheroids in sufficient concentration due to partial degradation. 

Due to the increased stability of the D-peptide, higher concentrations seem to reach the tumor cells, as well as normal cells, causing increased effects on both cell types compared to the L-peptide. Nevertheless, specificity for cancer cells is still high, which leaves the opportunity to treat with lower peptide doses. Conclusively, 9D-RDP215 is a promising new agent to treat glioblastoma as well as melanoma metastases to the brain due to its increased proteolytical stability, activity and its ability to pass the BBB to induce significant levels of peptide-induced cell death on tumor spheroids. 

## 4. Materials and Methods

### 4.1. Peptides

C-terminally amidated peptides (see Table 1) derived from Lactoferricin were purchased from PolyPeptide Group (San Diego, CA, USA). A purity of higher than 96% for all peptides had been determined by RP-HPLC. Peptides stock solutions were prepared in acetic acid (0.1%, *v*/*v*) to an approximate concentration of 3 mg/mL and treated by ultrasonication for 15 min for better solubility. The peptide concentration was determined by measurement of UV-absorbance of tryptophan at a wavelength of 280 nm using a NanoDrop photometer (ND 1000, Peqlab, VWR International, Inc. Erlangen, Germany). All peptide stocks were stored at 4 °C till use.

### 4.2. Lipids

The phospholipids 1,2-palmitoyl-oleoyl-sn-glycero-3-phosphocholine (POPC), 1,2-di-palmitoyl-*sn*-glycero-3-phosphocholine (DPPC), 1,2-palmitoyl-oleoyl-sn-glycero-3-phosphoserine (POPS) and 1,2-dipalmitoyl-*sn*-glycero-3-phospho-L-serine (Na-salt) (DPPS), were purchased from Avanti Polar Lipids, Inc. (Alabaster, AL, USA). Lipid stock solutions were prepared at a concentration of 10 mg/mL. The zwitterionic phospholipids (POPC and DPPC) were dissolved in a mixture of chloroform/methanol (2:1, *v*/*v*). The negatively charged phospholipids (DPPS and POPS) were dissolved in a mixture of chloroform/methanol (9:1, *v*/*v*).

### 4.3. Cell Lines and Cell Culture

The human malignant melanoma cell line (A375) and the human glioblastoma cell line LN-229 were purchased from ATCC (American Type Culture Collection, Manassas, VA, USA) and cultured in Dulbecco’s Modified Eagle Medium DMEM with GlutaMAXTM (Gibco^®^, Thermo Fisher Scientific, Waltham, MA, USA) supplemented with 10% fetal bovine serum FBS (Gibco^®^). Melanoma cell line from primary lesions SBcl-2 (kindly provided by Dr. Meenhard Herlyn, the Wistar Institute, Philadelphia, PA, USA) was maintained in RPMI 1640 medium with GlutaMAX™ (Gibco^®^, Thermo Fisher Scientific, USA) supplemented with 10% FBS (fetal bovine serum; Gibco^®^). The glioblastoma cell line U-87 mg, purchased from CSL (Cell Line Service, Eppelheim, Germany), was cultured in Minimum Essential Media with GlutaMAX™ (MEM; Gibco^®^, Thermo Fisher Scientific, Waltham, MA, USA) supplemented with 10% FBS (Gibco^®^, Thermo Fisher Scientific, Waltham, MA, USA), 0.1 mM non-essential amino acids (NEAA; Gibco^®^, Thermo Fisher Scientific, Waltham, MA, USA) and 1.0 mM sodium pyruvate (Gibco^®^). Normal human dermal fibroblasts NHDF (PromoCell Inc., Heidelberg, Germany) were used as non-neoplastic controls. NHDF were cultured in Fibroblast Growth Medium 2 (PromoCell Inc., Heidelberg, Germany). The Madin-Darby canine kidney cells MDCKII (hMDR1 KI) were purchased from Sigma-Aldrich (Sigma-Aldrich Co. LLC. St. Louis, MO, USA). The MDCKII cells are a subclone from the heterogenous parent MDCK line containing a canine MDR1 knockout and a human MDR1 knockin (MDCKII hMDR1 KI). The MDCKII (hMDR1 KI) cell line was cultured in Minimum Essential Medium with GlutaMAXTM, without HEPES and sodium pyruvate and with phenol red (MEM, Gibco^®^, Thermo Fisher Scientific, Waltham, MA, USA) supplemented with 10% fetal bovine serum. All cells were cultivated in an atmosphere with 5% CO2 at 37 °C. At 90% confluence, cells were passaged with accutase (Gibco^®^, Thermo Fisher Scientific, Waltham, MA, USA). NHDF cells were used at low passages (2–8) only.

For fluorescence microscopy studies, cells were seeded on Ibidi µ-Slide 8 wells (Martinsried, Munich, Germany).

### 4.4. Liposomal Model Studies

#### 4.4.1. Liposome Preparation

Appropriate amounts of respective phospholipid stock solution were dried under a stream of nitrogen and stored in vacuum overnight to completely remove organic solvents. The dry lipid film was then dispersed in phosphate-buffered saline (PBS, 20 mM NaPi, 130 mM NaCl, pH 7.4) and hydrated at a temperature well above the gel-to-fluid phase transition of the respective phospholipid under intermittent vigorous vortex-mixing. The lipid concentration was 0.1 weight% for calorimetric experiments. Hydration was carried out in the presence or absence of peptides at a lipid-to-peptide molar ratio of 25:1, 50:1 and 100:1. DPPC was hydrated at 50 °C, DPPS was hydrated at 65 °C, and POPS was hydrated at 30 °C. The lipid films were hydrated at respective temperatures for two hours while vortexing every 15 min for one minute. The fully hydrated samples were stored for at least 1 h at room temperature until measurement.

For leakage experiments, POPS and POPC lipid films were hydrated in 1 mL of ANTS/DPX (8-aminonaphthalene-1,3,6-trisulfonic acid/p-xylene-bis-pyridinium bromide) buffer following the protocols described above. Subsequently, the dispersions were extruded 20 times through a 100 nm Whatman^®^ NucleporeTM Track-Etched Membrane (Sigma-Aldrich Co. LLC), using a Mini-Extruder of Avanti Polar Lipids Inc. to obtain LUVs. Unilamellarity and size were tested by dynamic light scattering using Zetasizer Nano ZSP (Malvern Instruments, Herrenberg, Germany). The ANTS/DPX containing vesicles were separated from free ANTS/DPX by exclusion chromatography using a column filled with Sephadex™ G-75 (Amesham Biosciences) fine gel swollen in an isosmotic buffer (10 mM HEPES, 140 mM NaCl, pH 7.4). The void volume fractions were collected, and the phospholipid concentration was determined by phosphate analysis [76,77]. The liposomes containing ANTS/DPX were stored in the dark at room temperature.

#### 4.4.2. Circular Dichroism (CD)

CD measurements for secondary structure estimation were performed on a Jasco J 715 Spectropolarimeter (Jasco, Gross-Umstadt, Germany) at room temperature using quartz cuvettes with an optical path length of 0.02 cm. The CD spectra were measured between 260 nm and 180 nm with a 0.2 nm step resolution. To improve accuracy, 3 scans were accumulated and averaged. The peptides were dissolved in 20 mM phosphate-buffered saline (PBS) (20 mM NaPi, without NaCl, pH 7.4). Spectra were measured in the absence and presence of 20 mM POPS, 20 mM POPC and 0.5 µM Dodecylphosphocholine (DPC) (Avanti Polar Lipids, Alabaster, USA) and 0.5 µM sodium dodecyl sulfate (SDS) (Sigma Life Science, Darmstadt, Germany), with POPS and SDS mimicking cancer, POPC and DPC healthy mammalian membranes, respectively. Liposomes were prepared as described above. The respective peptide to surfactant molar ratios were 25:1. The background signals were subtracted after the measurements. Percentage secondary structure calculations were conducted using Spectra Secondary structure estimation [78].

#### 4.4.3. ANTS/DPX Leakage Experiments

The leakage of aqueous contents from POPS and POPC liposomes was determined using the 8-aminonaphthalene-1,3,6-trisulfonic acid/p-xylene-bis pyridinium bromide (ANTS/DPX) assay. The liposome preparation was performed as described above.

The leakage of aqueous contents from POPS and POPC liposomes was determined using the Cary Eclipse fluorescence spectrophotometer of Varian (Salt Lake City, UT, USA). Aliquots of LUVs were diluted with the isosmotic buffer to a final lipid concentration of 50 µM. Fluorescence spectra were recorded at 37 °C using an excitation wavelength of 360 nm, an emission wavelength of 530 nm and a slit width of 10 nm for both excitation and emission monochromators. Fluorescence emission was recorded as a function of time before and after the addition of incremental amounts of a peptide. The fluorescence increase due to leakage and subsequent dilution of quenched dye was measured after the addition of peptides. Peptides were added to final concentrations of 2, 4 and 8 µM, corresponding to peptide-to-lipid molar ratios of 1:25, 1:12.5 and 1:6.25, respectively. Data are presented in terms of fluorescence intensity (*I_F_*) and were calculated using Equation (1):

(1) $$I_{F}=\frac{\left(F-F_{0}\right)}{\left(F_{max}-F_{0}\right)}$$

where *F* is the measured fluorescence, *F*_0_ the initial fluorescence without peptide and *F_max_* the fluorescence corresponding to 100% leakage gained by addition of 1% Triton X-100.

#### 4.4.4. Differential Scanning Calorimetry

Differential Scanning Calorimetry (DSC) measurements were performed using a differential scanning calorimeter (VP-DSC) from MicroCal, Inc. (Northhampton, MA, USA). Heating scans were performed at a scan rate of 30 °C/h (pre-scan thermostating 30 min) with a final temperature of approximately 10 °C above the main transition temperature (T_m_) and cooling scans at the same scan rate (pre-scan thermostating 1 min) with a final temperature approximately 20 °C below T_m_. The heating/cooling cycle was performed three times. DPPC measurements were performed between 20 °C and 50 °C, DPPS measurements between 30 °C and 65 °C, and POPS measurements between 1 °C and 25 °C. To analyze the characteristics of the thermotropic phase behaviors, the MicroCal Origin Software (VP-DSC version) was used. After normalization of the phospholipid concentration and baseline adjustment, the peak areas were integrated to determine the enthalpy of the transition (ΔH). The phase transition temperature of the transition peak (T_m_) was determined as the maximum of the peak. The T_1/2_ (half-width) was defined as peak width at the half-height [79].

#### 4.4.5. Tryptophan/Acrylamide Quenching

Spectra of tryptophan fluorescence were measured at room temperature with a Cary Eclipse fluorescence spectrophotometer of Varian (Salt Lake City, UT, USA). The excitation wavelength was set to 280 nm, and the slid-width was 10 nm. The emission was recorded between 300 nm and 410 nm. 

Quenching of Tryptophan was carried out in the presence and absence of phospholipid liposomes (lipid-to-peptide ratio 25:1) using 0.1, 0.4 and 0.7 M acrylamide. The data were analyzed according to the Stern–Volmer equation (Equation (2)):

(2) $$\frac{F_{0}}{F}=1+K_{SV}\times \left[Q\right]$$

where *F*_0_ and *F* represent the fluorescence emission intensities in the absence and presence of the quencher molecule (*Q*), and *K_SV_* is the Stern–Volmer quenching constant, which is a quantitative measure for the accessibility of tryptophan to acrylamide [80].

### 4.5. Ex Vitro Studies

#### Stability Study

Peptide stability was tested in the presence of fetal bovine serum (FBS, Gibco^®^) and human serum (HS, Sigma-Aldrich, Deisenhofen, Germany). Therefore 50 µg of the peptide was mixed with 0 µL (0%), 4 µL (2%) and 20 µL (10%) pure FBS or HS and refilled with PBS buffer to a total volume of 200 µL and incubated for 1 day, 6 days and 7 days at 37 °C. Sample aliquots of 10 µL were taken before incubation (0 h) and after 1 h, 8 h, 24 h, 2 days and 7 days of incubation. The aliquots (10 µL) were immediately mixed with 10 µL 2 × loading sample buffer and stored at −20 °C. The collected samples of the stability studies were analyzed by SDS- polyacrylamide gel electrophoresis (BioRad, Mini-PROTEAN Tetra System). As a standard, the Ultra-low Range Molecular Weight Marker M3546 (Sigma-Aldrich, Deisenhofen, Germany) was used. Before use, the collected samples of the stability studies (10 µL aliquot + 10 µL 2 xLD), as well as the Ultra-Low Molecular Weight marker (ULMW), were brought to room temperature. A concentration series in the range of 0.5 µg, 1 µg and 2.5 µg peptide per 10 µL sample volume was prepared in PBS for each peptide. In total, 10 µL of the resulting samples (0.5 µg, 1 µg and 2.5 µg peptide) were mixed with 10 µL 2 × loading buffer. Afterwards, all samples (collected samples, ULMW, concentration series) were incubated at 95 °C for 10 min.

### 4.6. In Vitro Experiments

#### 4.6.1. Toxicity Assay—Propidium Iodide-Uptake Assay

To determine peptide-induced cell death, cells were resuspended in respective media (see Section 2.2) and diluted to a concentration of 1 × 10^6^ cells/mL. 100 µL Aliquots (10^5^ cells) were incubated with different peptide concentrations (5 µM to 100 µM) for up to 8 h in the presence of propidium iodide PI (2 µL/10^5^ cells of 50 µg/mL, Molecular Probes Inc., Eugene, OR, USA) at room temperature in black 96-well plates. PI-uptake was measured after 0, 1, 2, 4 and 8 h using the GloMax^®^ Multi+ Detection System (Promega, Madison, WI, USA). Cytotoxicity was calculated from the percentage of PI-positive cells in media alone (P_0_) and in the presence of peptide (P_X_) (see Equation (3)). Triton-X-100 was used to determine 100% of PI-positive cells (P_100_).

(3) $$\%PI-uptake=100\times \frac{\left(P_{X}-P_{0}\right)}{\left(P_{100}-P_{0}\right)}$$

Excitation and emission wavelengths were 536 nm and 617 nm, respectively. The cytotoxicity of the peptides was further investigated in the presence of different serum types and amounts, as well as in the presence of protease inhibition.

#### 4.6.2. Influence of Serum on Peptide Activity

The standard growth medium used for cell line A375 was DMEM containing 10% fetal bovine serum (FBS). Before the study of peptide-induced cytotoxicity, cells were diluted to 10^5^ cells/100 µL in DMEM containing 2% FBS. To investigate if the components in human serum (HS, from human male AB plasma, Sigma-Aldrich, St. Louis, MO, USA) have any different influence on the activity of the peptides, the cytotoxicity assay was also performed in DMEM containing 10% HS, as well as DMEM containing 2% HS. Before cytotoxicity studies in HS, cells were grown in a standard growth medium (DMEM with 10% FBS).

In the case of the protease inhibitor, 1µL of a 100 × protease inhibitor cocktail (Promega, Madison, WI, USA) was added to the diluted cell suspension (10^5^ cells/100 µL) before performing the cytotoxicity assay. The protease inhibitor cocktail was dissolved in 500 µL DMSO and stored at 4 °C.

#### 4.6.3. Apoptosis-/Necrosis Assay

To assess whether peptides induce apoptosis or necrosis, the RealTime-Glo™ Annexin V Apoptosis (luminescence) and Necrosis (fluorescence) Assay from Promega (Promega, Madison, WI, USA) was used. Briefly, cells were seeded at 10^5^ cells/100 µL in a white 96-multiwell plate with clear bottom and incubated overnight at 5% CO_2_ and 37 °C. The reagent stock solution (1000 X) was diluted in DMEM without phenol red containing 10% FBS, according to the manufacturer’s protocol. Before the measurement, 100 µL of the reagent solution was added to the cell suspension. For the fluorophore, excitation and emission wavelengths were 490 nm and 525 nm, respectively. Before the peptide addition, the signals at time zero were measured with the Glomax Multi+ detection system (Promega, Madison, WI, USA). Thereupon, the cells were treated with peptides at different concentrations (0 µM, 2 µM, 5 µM, 10 µM and 20 µM). Apoptosis and necrosis were measured every 30 min for 6 h. 

#### 4.6.4. Caspase-3/7 Assay

Peptide induced caspase-3/7 activity in the melanoma cell line A375 and the glioblastoma cell line LN-229 was determined using the Caspase-Glo^®^ 3/7 Assay (Promega, Madison, WI, USA). A total of 2 × 10^5^ cells/mL were seeded in white 96-well plates with clear bottom and grown for 48 h at 37 °C (5% CO_2_). Peptides were added to different concentrations for 2–4 h. Caspase solution was added to a final 1:1 ratio for 30 min at 37 °C. Luminescence intensity was recorded using the Glomax Multi+ detection system (Promega, Madison, WI, USA). Caspase-3/7 activity was calculated as a multiple of untreated cells.

#### 4.6.5. 3D Cell Culture—Generation of Multicellular Tumor Spheroids (MCTS)

Human glioblastoma LN-229 cells were used for experiments with MCTS. The glioblastoma cells were seeded at 10^4^ cells per 100 µL DMEM medium with 10% FBS into black Corning^®^ 96-well Spheroid Microplates with a round clear bottom (Ultra-Low Attachment surface) from Sigma-Aldrich (Deisenhofen, Germany) and grown for 5 days. 

#### 4.6.6. Fluorescence Microscopy: PI-Uptake of MCTS

The glioblastoma MCTS (LN-229) were grown as described above. For fluorescence microscopy, PI staining was used to determine the toxicity of the peptides implied by induction of membrane damage and cell death. Therefore, the MCTS were treated with peptides in different concentrations (2 µM, 5 µM, 10 µM and 20 µM) for 24 h. Then MCTS were harvested using Corning^®^ DeckWorks low binding pipet tips from Sigma-Aldrich (Deisenhofen, Germany), released onto ibidi 60 µ-Dish with glass bottom (Martinsried, Munich, Germany) and stained with 2 µL of a 50 µg/µL PI solution (in DPBS buffer) per spheroid. The experiments were performed with a Leica DMI6000 B with IMC in combination with a Leica DFC360 FX camera and AF 6000 software (Leica Microsystems, Vienna, Austria). For microscopic inspection, the excitation wavelength for PI was set to 538 nm, and the emission wavelength was set to 617 nm.

#### 4.6.7. 3D-Viability Assay

The CellTiter-Glo^®^ 3D-Cell Viability Assay from Promega (Promega, Madison, WI, USA) was used to determine the cell viability in 3D glioblastoma (LN-229) MCTS. The 3D assay reagent measures ATP as an indicator of viability and generates a detectable luminescent readout. For the assay, the spheroids were cultivated and seeded as previously described and treated with peptides for 48 h. In addition, a positive control was treated with 2 µL of Triton-X-100 (10%) representing 0% of cell viability (V_0_). The luminescence signal was measured using the Glomax Multi+ detection system (Promega, Madison, WI, USA) at room temperature for peptide concentrations from 0–100 µM. The plate was shaken vigorously for 5 min and afterwards incubated for 25 min. The viability was calculated from the percentage of viable cells without peptide (V_100_) and in the presence of peptide (V_X_) (Equation (4)).

(4) $$\%viability=100\times \frac{\left(V_{X}-V_{0}\right)}{\left(V_{100}-V_{0}\right)}$$

#### 4.6.8. Stimulation of the Blood-Brain Barrier (BBB)

The three-dimensional glioma blood-brain-barrier model [58] was used to investigate the permeability and cytotoxicity of peptides on glioblastoma spheroids. MDCKII (hMDR1 KI) cells were cultured as previously described and seeded into Corning^®^ HTS 96-well Transwell (Corning HTS Transwell^®^- 96 Permeable Support 0.4 µm PET Membrane, 0.143 cm^2^) purchased from Sigma-Aldrich (Deisenhofen, Germany) at 1.43 × 10^4^ cells per 100 µL of the respective medium. 200 µL of the medium was added to each well of the reservoir plate. LN-229 cells were cultured as previously described and seeded at 1 × 10^4^ per 100 µL of the respective medium into Corning^®^ 96-well Spheroid Microplates with a round clear bottom (Ultra-Low Attachment surface) from Sigma-Aldrich (Deisenhofen, Germany). Both cell lines were grown for 5 days. After 5 days, 100 µL medium (200 µL total medium required) was added to the spheroids in the Corning^®^ 96-well Spheroid Microplates. Afterwards, the MDCKII (hMDR1 KI) and LN-229 cells were combined through a transfer of the Corning^®^ HTS 96-well Transwell insert onto the Corning^®^ 96-well Spheroid Microplates. Then, peptides in different concentrations (0 µM, 2 µM, 5 µM, 10 µM, 20 µM, 40 µM and 100 µM) were added to the Transwell insert. As a positive control, 2 µL Triton-X-100 (10%) was directly added to the spheroids. The cells were incubated for 48 h in an atmosphere with 5% CO_2_ at 37 °C.

BBB-model—3D-Viability assay: The 3D-cell viability was measured using CellTiter-Glo^®^ 3D Cell Viability Assay from Promega Glomax Multi+ detection system (Promega, Madison, WI, USA). After 48 h of incubation, the plate was equilibrated to room temperature (22–25 °C) for approximately 30 min. The Transwell insert was retransferred into the reservoir plate, and 100 µL of the medium in the Corning^®^ 96-well Spheroid Microplates was removed. The CellTiter-Glo^®^ 3D Reagent (Promega, Madison, WI, USA) was added, and luminescence was recorded as described above.

BBB-Model—Monolayer integrity: After the peptide study, the Transwell MDCKII (hMDR1 KI) cells were tested for monolayer integrity. Lucifer yellow from Sigma-Aldrich (Deisenhofen, Germany) was diluted to a final concentration of 0.02 mg/mL in Hanks’ Balanced Salt solution (HBBS) from Sigma-Aldrich (Deisenhofen, Germany), which is modified with sodium bicarbonate, without phenol red, calcium chloride and magnesium sulfate. A total of 150 µL of HBBS was added into a new black 96 micro-well plate (Fisher Scientific GmbH, Austria), serving as the basolateral receiver chamber. The complete medium was removed from the Transwell MDCKII (hMDR1 KI) cells, and the Transwell was transferred onto the black 96 micro-well plates containing the 150 µL HBBS. 100 µL of the Lucifer yellow was added to the Transwell and incubated for 60 min in an atmosphere with 5% CO_2_ at 37 °C. The Transwell was removed again, and fluorescence (RFU) in the basolateral receiver plate was measured at 485/535 nm using Promega Glomax Multi+ detection system (Promega, Madison, WI, USA). In addition, 150 µL of pure HBBS served as a negative control for 100% monolayer integrity and 150 µL of a mixture of HBSS and Lucifer yellow starting solution (LY_starting solution_) as a positive control, referring to 0% monolayer integrity. The percentage of rejection was calculated for all wells and their various peptides concentrations (LY_acceptor_) (Equation (5)). Wells with rejection over 5% were to be discarded.

(5) $$\%rejection=100\times \frac{RFU\;[LY_{acceptor}]}{RFU\left[LY_{starting\;solution}\right]}$$

### 4.7. Statistical Analysis

The studies were performed with defined stock solutions of lipids and peptides with purity ≥95%. Values are presented as the mean ± SEM. Model, cell culture and microscopy studies were repeated at least three times. For microscopy studies, a set of data being representative of the respective results is shown.

## Figures and Tables

**Table 1:** Amino acid sequence, molecular weight and net charge of RDP215 and 9D-RD215. Lowercase letters represent D-amino acids.

Peptide.	Sequence	Amino Acids	Molecular Weight (g/mol)	Net Charge
R-DIM-P-LF11-215 (RDP215)	H-FWRIRIRR-P-RRIRIRWF-NH_2_	17	2483.03	+9
9D-R-DIM-P-LF11-215 (9D-RDP215)	H-fWrIrIrr-P-rrIrIrWf-NH_2_	17	2483.03	+9

**Table 2:** The mean percentage of ANTS/DPX leakage of POPS liposomes in the presence of RDP215 and 9D-RDP215 at different peptide concentrations (0.5–8 µM).

	ANTS/DPX Leakage (%)	
peptide Concentration (µM)	*RDP215*	*9D-RDP215*
0.5	7.9 ± 0.9	2.6 ± 0.3
1.0	14.2 ± 1.9	3.2 ± 0.7
2.0	31.5 ± 4.8	22.3 ± 2.5
4.0	83.3 ± 7.1	81.2 ± 0.5
8.0	99.2 ± 0.7	± 0.6

**Table 3:** Thermodynamic parameters of DPPS, POPS and DPPC liposomes in the absence and presence of RDP215 and 9D-RDP215 with different molar lipid-to-peptide ratios (100:1; 50:1; 25:1). DSC data analyzed with MicroCal Origin Software (VP-DSC version) are representative results of three independent experiments. Deconvolution was used for analysis of data with overlapping phase transitions.

	ΔH_pre or *_ [kcal/mol]	T_pre or *_ (°C)	ΔH_m 1/2/3_ *(Total)* (kcal/mol)	T_m 1/2/3_ (°C)	T_1/2 1/2/3_ (°C)
DPPS	-	-	10.8	52.9	0.34
+RDP215 100:1	0.1 (*)	39.1 (*)	2.2/10.3 *(12.5)*	47.5/52.9	1.60/0.36
+RDP215 50:1	0.4 (*)	39.5 (*)	4.8/1.2/4.2 *(10.2)*	48.5/49.0/52.8	1.49/0.66/0.39
+RDP215 25:1	0.9 (*)	39.5 (*)	5.9/1.2/2.4 *(9.5)*	48.6/49.2/52.9	1.62/1.13/0.34
+9D-RDP215 100:1	0.9 (*)	39.4 (*)	0.6/9.5 *(10.1)*	51.3/52.9	1.25/1.34/0.35
+9D-RDP215 50:1	1.8 (*)	39.7 (*)	0.6/6.9 *(7.5)*	50.5/52.9	0.70/0.93/0.57
+9D-RDP215 25:1	3.9 (*)	40.0 (*)	0.7/2.8/0.2 *(3.7)*	50.5/52.4/52.8	0.32/2.52
POPS	-	-	5.3	11.8	1.76
+RDP215 100:1	-	-	0.7/3.4 *(4.1)*	8.1/11.7	1.95/1.84
+RDP215 50:1	-	-	0.9/2.2 *(3.1)*	8.3/11.8	2.31/1.83
+RDP215 25:1	-	-	0.1/0.7 *(0.8)*	8.2/11.8	1.35/1.72
+9D-RDP215 100:1	-	-	3.9	11.6	1.73
+9D-RDP215 50:1	-	-	2.5	11.8	1.78
+9D-RDP215 25:1	-	-	0.5	11.7	1.91
DPPC	1.9	35.9	10.3	41.6	0.14
+RDP215 100:1	1.6	35.8	9.8	41.6	0.14
+RDP215 50:1	1.6	35.6	9.9	41.6	0.15
+RDP215 25:1	1.3	35.6	8.9	41.6	0.14
+9D-RDP215 100:1	1.7	35.9	10.9	41.6	0.16
+9D-RDP215 50:1	2.0	35.8	12.0	41.6	0.15
+9D-RDP215 25:1	2.0	35.8	11.1	41.6	0.14

* Additional transition, potentially gel ordered to gel disordered; 1/2/3 data analyzed for multiple peaks.

**Table 4:** A comparison of the mean values of Stern–Volmer constants K_SV_ and maxima of emission wavelengths Δλ_em, max_ of RDP215 and 9D-RDP215 in PBS with the values determined in the presence of cancer mimics DPPS and POPS and the non-cancer mimic DPPC with a lipid-to-peptide ratio of 25:1. The mean values are given with standard deviation calculated from three independent experiments.

	RDP215		9D-RDP215	
	λ _em,max_ [nm]	K_SV_ (M^−1^)	λ _em,max_ [nm]	K_SV_ (M^−1^)
PBS	356 ± 0	24.7 ± 5.4	354 ± 0.5	27.0 ± 2.8
	Δλ_em,max_ [nm]	K_SV_ (M^−1^)	Δλ_em,max_ [nm]	K_SV_ (M^−1^)
DPPS	−20 ± 0	4.0 ± 0.3	−14 ± 1	3.9 ± 0.5
POPS	−21 ± 1	4.8 ± 1.2	−16 ± 1	4.1 ± 0.7
DPPC	−1 ± 0	19.5 ± 1.0	−1 ± 0	21.7 ± 0.4

**Table 5:** A comparison of LC_50_ values (µM) of the melanoma cell line A375 and normal human dermal fibroblasts NHDF determined by PI-uptake upon 4 and 8 h of peptide treatment.

A375	LC_50_ (µM) after 4 h	Specificity NHDF/A375	LC_50_ (µM) after 8 h	Specificity NHDF/A375
RDP215	17.1 ± 1.5	5.8	3.8 ± 0.3	18.0
9D-RDP215	7.0 ± 1.0	8.4	1.4 ± 0.1	13.5
SBcl-2			LC_50_ (µM) after 8 h	Specificity NHDF/SBcl-2
RDP215			4.7 ± 0.4	14.5
9D-RDP215			1.8 ± 0.1	10.5
NHDF	LC_50_ (µM) after 4 h		LC_50_ (µM) after 8 h	
RDP215	99.1 ± 0.1		68.2 ± 1.8	
9D-RDP215	58.8 ± 4.2		18.9 ± 1.0	

**Table 6:** A comparison of LC_50_ values (µM) of cell lines LN-229 glioblastoma and normal human dermal fibroblasts NHDF determined by PI-uptake upon 8 h of peptide treatment.

LN-229	LC_50_ (µM) after 8 h	Specificity NHDF/LN-229
RDP215	1.7 ± 0.1	40.1
9D-RDP215	1.4 ± 5.1	13.5
U-87 mg	LC_50_ (µM) after 8 h	Specificity NHDF/U-87 mg
RDP215	3.0 ± 0.1	22.7
9D-RDP215	1.6 ± 0.1	11.8
NHDF	LC_50_ (µM) after 8 h	
RDP215	68.2 ± 1.8	
9D-RDP215	18.9 ± 1.0	

## Data Availability

The data presented in this study are available on request from the corresponding authors. They are not publicly available due to non-standard formats.

## Supplementary Materials

The following are available online at https://www.mdpi.com/article/10.3390/ijms22168469/s1.
