# Supplementary material for: Effect of L- to D-Amino Acid Substitution on Stability and Activity of Antitumor Peptide RDP215 against Human Melanoma and Glioblastoma
Source: Int J Mol Sci. 2021 Aug 6;22(16):8469. doi: 10.3390/ijms22168469 (PMC8395111; doi:10.3390/ijms22168469)
Supplement: Supplementary file 1 [file ijms-22-08469-s001.zip › Figure S2.pdf]

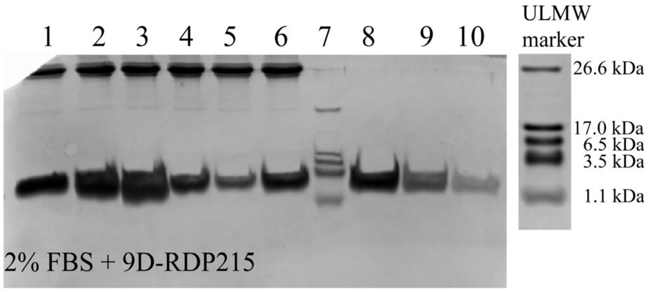

Figure S2. SDS-polyacrylamide gel electrophoresis of stability studies of 9D-RDP215 in presence of 2% fetal bovine serum (FBS). Peptide 9D-RDP215 (2.5 μg) was incubated at 37°C for up to 7 days in presence of 2% FBS, respectively. Lane 1-6: 2.5 μg respective peptide was incubated in presence of 2% FBS for 0 h (lane 1), 1 h (lane 2), 8 h (lane 3), 24 h (lane 4), 2 days (lane 5), and 7 days (lane 6). Molecular weights can be estimated from the ULMW marker (lane 7), fragment sizes are given on the right. Lane 8-10: 2.5 μg, 1 μg, and 0.5 μg respective peptide control. Presented data are representative for results of two independent measurements.
