# Supplementary material for: Effect of L- to D-Amino Acid Substitution on Stability and Activity of Antitumor Peptide RDP215 against Human Melanoma and Glioblastoma
Source: Int J Mol Sci. 2021 Aug 6;22(16):8469. doi: 10.3390/ijms22168469 (PMC8395111; doi:10.3390/ijms22168469)
Supplement: Supplementary file 1 [file ijms-22-08469-s001.zip › Figure S4_rev.pdf]

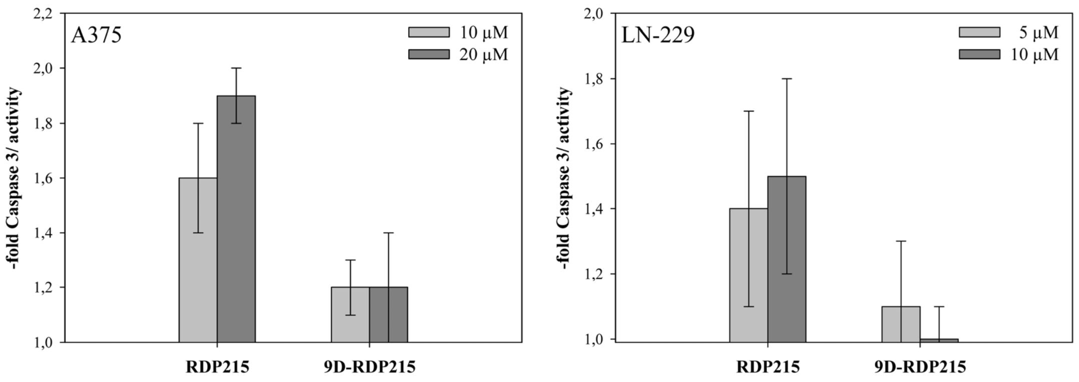

Figure S4: Left: Fold caspase-3/7 activity of melanoma cell line A375 after 4 h of incubation with 10 and 20 µM of peptide RDP215 and 9D-RDP215 indicating increased killing by early apoptosis by RDP215 compared to the L-peptide. Right: Fold caspase-3/7 activity of glioblastoma cell line LN-229 after 2 h of incubation with 5 and 10 µM of peptide RDP215 and 9D-RDP215 only indicating killing by early apoptosis by RDP215. Mean values represent data of at least three independent experiments.
