# Supplementary material for: Effect of L- to D-Amino Acid Substitution on Stability and Activity of Antitumor Peptide RDP215 against Human Melanoma and Glioblastoma
Source: Int J Mol Sci. 2021 Aug 6;22(16):8469. doi: 10.3390/ijms22168469 (PMC8395111; doi:10.3390/ijms22168469)
Supplement: Supplementary file 1 [file ijms-22-08469-s001.zip › Figure S5_rev.pdf]

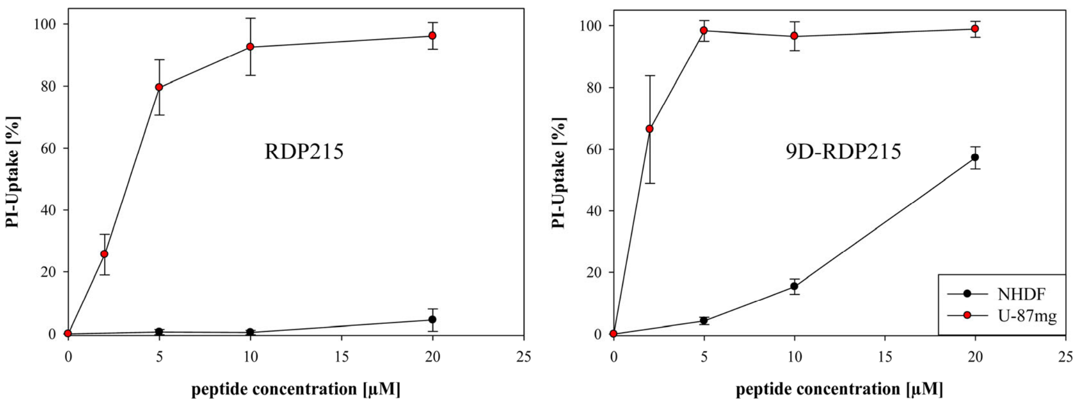

Figure S5. Cytotoxicity of RDP215 and 9D-RDP215 on glioblastoma cells U-87mg and healthy cells NHDF. Cell death was determined by PI-uptake (%) in presence of 2 µM, 5 µM, 10 µM and 20 µM peptide concentration after 8 hours of incubation. With U-87mg, RDP215 already displays high peptide induced cell death up to 80% at 5 µM. Within the studied concentration range RDP215 exhibits no harm on NHDF cells. 9D-RDP215 shows increased antitumor and non-tumor toxicity, though still with high specificity. E.g., at 5 µM the D-peptide exhibits about 30-fold higher killing of cancer cells than normal cells. Mean values represent data of at least three independent experiments.
